# Matrine as a multi-disease regulator of ferroptosis: mechanisms, therapeutic applications, and toxicity management

**DOI:** 10.3389/fphar.2026.1860965

**Published:** 2026-06-25

**Authors:** Yuhan Lu, Jianrong Ye

**Affiliations:** Department of Anesthesiology, The First Affiliated Hospital of Xinjiang Medical University, Urumqi, China

**Keywords:** ferroptosis, iron metabolism, matrine, multi-target, toxicity

## Abstract

Matrine is the main active metabolite of *Sophora flavescens Ait*. Matrine is a traditional medicine with a long history of medicinal use. Modern pharmacological research confirms that matrine possesses a variety of pharmacological effects, including anti-inflammatory, immunomodulatory, and anti-parasitic activities. Ferroptosis is a form of programmed cell death characterized by iron accumulation and lipid peroxidation. Recent studies show that matrine achieves organ protection or therapeutic effects in various diseases by regulating the shared pathway of ferroptosis. This review explores the multi-target intervention role of matrine in cardiovascular diseases, central nervous system diseases, acute lung injury induced by severe acute pancreatitis, and cervical cancer through the mechanism of ferroptosis. By comparing and deeply analyzing different research findings, the review provides a theoretical basis for future research and potential drug development. It also integrates existing preclinical evidence and emphasizes the need for rigorous, multi-center, and large-scale clinical studies to accelerate the translation of matrine from the laboratory to clinical practice. The cross-disease therapeutic potential of matrine via ferroptosis regulation positions it as a candidate molecule linking the multi-target advantages of natural metabolites with modern precision medicine. However, its dose-dependent tissue toxicity necessitates that future research integrate derivative optimization, targeted delivery systems, and biomarker-based individualized dosing strategies to achieve a safe transition from laboratory to clinical practice.

## 1 Introduction

This review summarizes how matrine influences ferroptosis and explores its therapeutic value across disease models.

### 1.1 Mechanism and pathological effects of ferroptosis

Cell death is a critical part of life and a fundamental biological process that includes several complex and multifaceted forms, such as apoptosis, necrosis, and autophagy. In recent years, ferroptosis has attrached widespread attension as a distinct form of cell death. This form of cell death was first described in (Dixon et al., 2012). Unlike traditional modes of cell death, ferroptosis has unique molecular mechanisms and cellular characteristics: greatly increased intracellular iron levels promote chain reactions of lipid peroxidation, leading to cellular membrane damage and, ultimately, cell death (Ru et al., 2024). Ferroptosis has been shown to play a crucial role in pathological processes such as cardiovascular diseases, neurodegenerative diseases, tumors, and inflammatory diseases (Wu et al., 2021). Therefore, detailed studies of the mechanisms of ferroptosis and its regulatory factors are important for developing new therapeutic approaches for these diseases (Zhou et al., 2024).

### 1.2 Pharmacological effects and clinical application challenges of matrine

Natural products have long been an important source of drug development, and the discovery of many modern drugs has relied on extensive research on natural plant chemistry. Matrine is a quinolizidine alkaloid extracted from leguminous plants, primarily *Sophora flavescens Aiton* (Fabaceae/Leguminosae). The taxonomic identity of the source plant has been verified against the authoritative databases Plants of the World Online (POWO; https://powo.science.kew.org/taxon/urn:lsid:ipni.org:names) and the Medicinal Plant Names Services (MPNS; http://mpns.kew.org/mpns-portal/). It exhibits broad-spectrum, multi-pathway, and multi-target properties and has diverse pharmacological activities, including cardiovascular protection, anticancer, anti-inflammatory, antibacterial, and antiviral effects. Its mechanisms of action include inhibition of signaling pathways such as epidermal growth factor receptor (EGFR), nuclear factor kappa-light-chain-enhancer of activated B Cells (NF-κB), and p38 mitogen-activated protein kinase (p38 MAPK), activation of the Nrf2/HO-1 antioxidant axis, and regulation of the gut microbiota (Tao et al., 2024). Despite progress in matrine research, current studies still face several challenges (Sun et al., 2022).

To this end, future research should combine organ-like models with artificial intelligence-driven virtual screening, systematically build a multi-dimensional pharmacological network of matrine, and clarify its mechanism and targets in a variety of diseases, so as to provide a more solid theoretical basis for clinical applications. The preparation strategy and targeted delivery system need to be improved to overcome the key bottlenecks in the clinical application of matrine. The targeted delivery system can increase the concentration of matrine in the disease site, enhance the therapeutic effect, and reduce the exposure of normal tissue. This can minimize adverse reactions and improve the safety and effectiveness of clinical applications. For example, nanotechnology-based preparations can improve the *in vivo* stability and targeting of matrine and reduce its distribution in non-target tissues, thus reducing its toxicity. The formulation of individualized treatment plans based on biomarkers, as well as the precise adjustment of the dosage and administration plan of matrine according to the disease status and drug metabolic characteristics, are also important research directions in the future (Xu et al., 2022).

## 2 Bibliographic search strategy

The search work covered PubMed, Embase, Web of Science, and Cochrane Library databases, and the deadline was 12 April 2026. In order to ensure the breadth and accuracy of the search, we constructed a series of search terms. These search words focused on the core concepts, including “ferroptosis”, “matrine”, and key disease models or symptoms, such as “myocardial injury”, “atherosclerosis”, “experimental autoimmune encephalomyelitis”, “severe acute pancreatitis”, “acute lung injury”, “cervical cancer”, and “toxicity”. We used “ferroptosis regulation” as a unified mechanistic anchor to establish a three-dimensional integrative framework of “matrine intervention–ferroptosis–disease microenvironment,” thereby systematically connecting existing research summaries with clinical translation pathways.

In this narrative review, a rigorous and objective framework was employed. The inclusion boundary of this review is strictly limited to studies that report a regulatory effect of matrine on the ferroptosis pathway. Priority was given to data from randomized controlled trials (RCTs), systematic reviews, and meta-analyses published within the last 20 years, while seminal earlier work was also considered where high-impact clinical data were presented. We aimed to identify all high-quality studies to provide a solid evidence base for the clinical application of matrine and to guide future research directions. The screening process resulted in the removal of duplicate records (n = 124), studies irrelevant to the topic (n = 47), and non-English publications (n = 3). Ultimately, a total of 78 studies were included for final analysis in this review.

## 3 Ferroptosis

Ferroptosis is an iron-dependent, non-apoptotic cell death driven by lipid peroxidation. Its hallmarks include accumulation of lipid peroxides, increased labile iron, and downregulation of the antioxidant system, particularly the GPX4-GSH axis and the Nrf2-dependent antioxidant program. Key positive regulators include ACSL4 and TFR1; negative regulators include GPX4, SLC7A11/xCT, and FSP1. This review focuses on how matrine modulates these pathways in a disease-context-dependent manner.

### 3.1 Mechanism of ferroptosis: interactions of iron metabolism and oxidative stress

In cell biology, the interaction between iron metabolism and oxidative stress drives ferroptosis. This process affects the fate of cells under physiological and pathological conditions (Jiang et al., 2021). Intracellular iron ion accumulation is the basic biochemical feature of ferroptosis. Ferrous ions (Fe^2^+) play a dual role in cells. On the one hand, they participate in a variety of biochemical reactions to maintain normal cell function. On the other hand, the accumulated iron ions trigger the excessive production of reactive oxygen (ROS) through the Fenton reaction. These reactive oxygens will attack polyunsaturated fatty acids (PUFA) in cell membranes, triggering a lipid peroxidation chain reaction. This process destroys the integrity of the cell membrane and damages the cell function.

**FIGURE 1:**
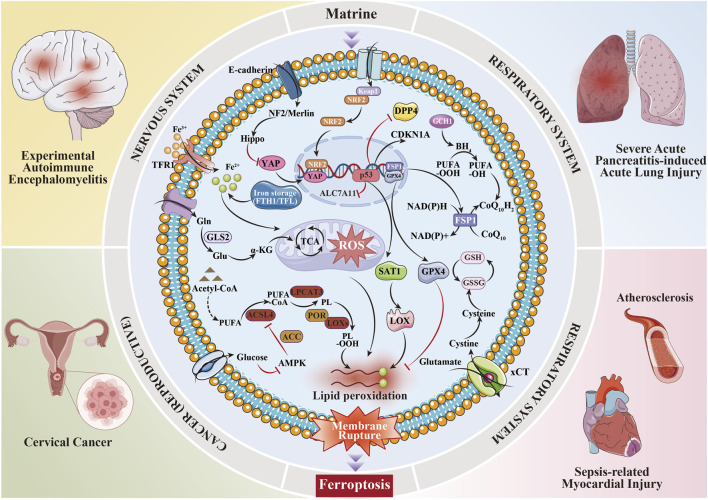
Ferroptosis is initiated by the synthesis of polyunsaturated fatty acid-containing phospholipids, subsequent lipid peroxidation, and iron-dependent toxicity. This figure was created with BioRender.com.

Ferroptosis pathways are complex, including multiple key factors and signal transduction routes (Tang et al., 2021). In the GPX4 pathway, GPX4 is a core negative regulator whose activity depends on the supply of GSH. Inducers such as RSL3 inhibit GPX4 activity and disrupt intracellular redox balance, thereby triggering ferroptosis. In contrast, the Nrf2 signaling pathway maintains intracellular redox balance and inhibits ferroptosis by regulating the expression of antioxidant genes. When Nrf2 signaling is activated, cellular antioxidant capacity is enhanced, which can reduce susceptibility to ferroptosis. Meanwhile, when the Nrf2 signaling pathway is inhibited, cells may more easily undergo ferroptosis. In the ACSL4 pathway, ACSL4 is a critical positive regulator of ferroptosis. High expression of ACSL4 is closely related to higher cellular vulnerability to ferroptosis. It promotes the activation of long-chain fatty acids and thus increases the substrates for lipid peroxidation, thereby aggravating lipid peroxidation and facilitating ferroptosis (Doll et al., 2017). Meanwhile, ACSL4 may regulate susceptibility to lipid peroxidation balance. By influencing lipid metabolism, it changes the cell membrane lipid composition, and cells are more susceptible to ferroptosis. In iron metabolism-related pathways, ferritin degradation and dysfunction of ferroportin are essential in regulating intracellular iron levels. Ferritin degradation releases iron ions and increases the intracellular iron level. Similarly, dysfunction of ferroportin prevents iron efflux, further enhancing intracellular iron accumulation. Abnormal regulation of these pathways can disrupt iron homeostasis, triggering ferroptosis. In the context of matrine research, three key nodes in this process—GPX4 antioxidant defense, Nrf2 transcriptional regulation, and ACSL4 lipid metabolism—have been confirmed as direct or indirect targets of matrine. Specifically, matrine inhibits pathological ferroptosis by upregulating the SLC7A11/GPX4 axis, or induces ferroptosis in tumor cells by activating the Piezo1/Ca^2+^ cascade to suppress GPX4, whereas at certain doses it triggers ferroptosis in normal tissues by inhibiting Nrf2 nuclear translocation. Given that matrine exhibits multi-directional regulatory potential on ferroptosis, future research should prioritize the use of targeted metabolomics and chemical proteomics to identify the binding modes of matrine with GPX4, ACSL4, Piezo1, or Nrf2. As displayed in Figure 1, the mechanism of action underlying ferroptosis is depicted.

## 4 Matrine: translational progress of a traditional Chinese medicine monomeric metabolite

Matrine is a major alkaloid derived from *S. flavescens Ait.* Its structural features contribute to diverse pharmacological activities and have motivated continued investigation of its therapeutic potential.

### 4.1 Chemical structure of matrine: structural characteristics of quinolizidine alkaloids

Matrine is a kind of quinolicidine alkaloid with the molecular formula C15H24N2O. Its structure contains a ketone group and an amino group, forming a quadricyclic skeleton (Li et al., 2025a). The multi-ring skeleton gives a clear three-dimensional conformation and relative hydrophobicity. These properties may help it interact with biological targets such as ion channels and enzyme active sites (Zhang et al., 2020). These structural characteristics can explain the extensive biological activity of matrin to a certain extent. The commonly used extraction method is solvent extraction. This method is low-cost and easy to operate, so it is widely used. However, the purity of its extract is limited (for example, 60%–70%) and there may be residual solvents.

Supercritical CO2 fluid extraction can achieve the selective enrichment of matrine by regulating temperature and pressure. The purity obtained by this method can reach more than 90%, and there is no organic solvent residue (Xu et al., 2022). However, the high cost of equipment is a major obstacle. Ultrasonic or microwave-assisted extraction can destroy the plant cell wall, thus improving the extraction efficiency. The purification of large-pore resin can further improve the purity and overall preparation efficiency of matrine (Li et al., 2021).

### 4.2 Matrine derivatives: strategic scaffold diversification

In recent years, structural modification to develop derivatives with higher activity and better druggability has become a significant research direction. In the field of anticancer research, a series of matrine derivatives have demonstrated significantly enhanced cytotoxic activity. For instance, the matrine-derived metabolite MASM can inhibit the proliferation and induce differentiation of hepatocellular carcinoma (HCC) cells and their cancer stem-like cells (EpCAM + cells) by suppressing multiple signaling pathways, including PI3K/AKT/mTOR and AKT/GSK3β/β-catenin (Liu et al., 2017). Similarly, WM130 preferentially inhibits hepatic cancer stem-like cells, a mechanism also associated with the downregulation of the AKT/GSK3β/β-catenin pathway (Ni et al., 2016). Other derivatives, such as WM622, function by inhibiting the PI3K/AKT signaling pathways (Sun et al., 2018), while YYJ18 induces apoptosis in nasopharyngeal carcinoma cells by targeting MAPK and PI3K/Akt pathways (Xie et al., 2014). Furthermore, derivatives like YF-18 inhibit lung cancer cells by down-regulating Skp2 (Wu et al., 2017), and Sophflarine A suppresses non-small cell lung cancer by inducing ROS-mediated pyroptosis and autophagy (Luo et al., 2023b). Structure-activity relationship studies indicate that introducing aromatic rings, heterocycles (e.g., thiophene, pyrazole, indole), or specific functional groups (e.g., acylhydrazone, dithiocarbamate) onto the parent nucleus can markedly improve antiproliferative activity (Ni et al., 2023). Some derivatives are designed as dual inhibitors targeting Hsp90, topoisomerase I or PARP, which reveals a new mechanism of action (Ou et al., 2025).

Matrine derivatives also have a significant effect on anti-organ fibrosis. MD-1 and WM130 improve liver fibrosis by blocking EGFR and inhibiting the phosphorylation of Akt in hepatic astrocytes (HSCs) (Qian et al., 2015). MASM has also been confirmed to have anti-fibrosis effect in the liver disease model. In the pulmonary fibrosis model, the derivative 3f significantly weakens fibrosis remodeling by inhibiting the TGF-β/Smad pathway (Li et al., 2019). In addition to tumors and fibrosis, matrine derivatives also have prospects for anti-inflammatory, neuroprotective and antiviral treatment. MASM alleviated excessive inflammation and tissue damage in mouse sepsis, experimental autoimmune encephalomyelitis and collagen-induced arthritis models by double blocking the NF-κB and MAPK pathways (Fan et al., 2022). It can also reverse the depression-like behavior induced by LPS by restoring the inflammatory homeostasis, redox balance and autophagy flux in the hippocampic region (Li et al., 2025b). The newly isolated matrine alkaloids Sophflarines B-E has a neuroprotective effect, and its mechanism may be related to the activation of the Keap1-Nrf2/HO-1 axis (Luo et al., 2025). Antiviral activity analysis shows that some matrine derivatives can inhibit HIV-1 and tobacco leaf virus (Huang et al., 2018; Ni et al., 2022). Computer simulation predicts that matrine is also active against Monkeypox virus and Marburg virus (Islam et al., 2024). In crop protection, matrine derivatives serve as privileged leads for next-generation, eco-compatible pesticides (Ang et al., 2023; Cheng et al., 2023; Huang et al., 2024). The pharmacokinetics and safety research of matrine analogues is also advancing. Take MASM as an example, its oral bioavailability is higher than that of the parent alkaloid, the acute toxicity (LD50) is slightly improved, and it has measurable brain permeability (Li et al., 2024).

As shown in Table 1, systematic structural modification of matrine consistently amplifies its intrinsic bioactivity and unlocks novel indications. These integrated data sets delineate robust structure–activity relationships and furnish a mechanistically grounded blueprint for continued optimization and translational development of matrine-based agents (Rashid et al., 2019).

**TABLE 1:** Biological activities and mechanisms of matrine derivatives.

Disease/Pathological model	Compound (derivative)	Mechanism	Model	References (author and year)
Pain	6-Acyldecahydro [1,6]naphthyridine derivatives (17b, 17c)	Structure-activity relationship: Less hindered tertiary amine and lipophilic acyl group enhance potency; A/B rings of matrine not essential	*In* *vivo*	Kobashi et al. (2003)
Pain	1-Acyl-4-dialkylaminopiperidines	Structure-activity relationship: Amide group is essential for antinociceptive potency	*In* *vivo*	Kobashi et al. (2002)
Neuroprotection	Sophflarines B-E	Reducing cytokines (NO, TNF-α, IL-6); compound 2 activates Keap1-Nrf2/HO-1 pathway	*In* *vitro*	Luo et al. (2025)
Inflammation	7-Epi-sophoramine; aluminum sophaloseedline T	Inhibiting pro-inflammatory factors (TNF-α, IL-6, IL-1β) and protein expression (iNOS, COX-2)	*In vitro*	Luo et al. (2024)
Non-small Cell lung cancer	Sophflarine A (SFA is a novel 6/8/6/6 tetracyclic ring system constructed from 5,6-*seco*-matrine via an intramolecular hemiketal bridge)	Activation of NLRP3/caspase-1/GSDMD (pyroptosis); ↑ROS, blockade of PI3K/AKT/mTOR (autophagy); suppression of EMT.	*In vitro and* *in vivo*	Luo et al. (2023)
Radiation injury	MASM((6aS, 10S, 11aR, 11bR, 11 cS)-10-methylamino-dodecahydro-3a, 7a-diazabenzo (de) anthracene-8-thione)	Modulation of multiple signaling pathways (e.g., metabolic, cancer, MAPK pathways)	*In* *vivo*	Li et al. (2016)
Sepsis	MASM	Inhibition of NF-κB and MAPK pathways; action mediated partly by RPSS.	*In* *vitro and* *in vivo*	Xu et al. (2016a)
Dendritic Cell maturation	MASM	Inhibition of expression of CD80/CD86, cytokines (IL-12, TNF-α, IL-6, NO); inhibition of PI3K/Akt, MAPK, NF-κB pathways	*In* *vitro*	Xu et al. (2016b)
Rheumatoid arthritis	MASM	Inhibition of pro-inflammatory cytokines (TNF-α, IL-1β, IL-6) and MMPs; inhibition of MAPKs and NF-κB; induction of apoptosis via mitochondrial and Akt pathways	*In* *vitro and* *in vivo*	Zou et al. (2017)
Hepatocellular carcinoma	MASM	Induction of apoptosis (↓Bcl-2, ↑cleaved PARP); cell cycle arrest (↑p27, ↓Cyclin D1); suppression of PI3K/AKT/mTOR and AKT/GSK3β/β-catenin pathways	*In* *vitro and* *in vivo*	Liu et al. (2017)
Epithelial cancer	MASM	Induction of ROS-dependent apoptosis and autophagy; involvement of inhibition of Akt and activation of Erk/p38	*In* *vitro*	Zou et al. (2019)
Liver injury and fibrosis	MASM	Inhibition of infiltration of Gr1hi monocytes and MCP-1 expression/activity	*In* *vitro and* *in vivo*	Xu et al. (2019)
Hepatocellular carcinoma	MASM	Downregulation of stemness genes; inhibition of AKT/GSK3β/β-catenin signaling	*In* *vitro*	Sun et al. (2022)
Multiple sclerosis (EAE)	MASM	Inhibition of astrocyte reactivity (A1 formation), maintenance of astrocytic function, improvement of BBB function	*In* *vitro and* *in vivo*	Fan et al. (2022)
Depressive-like behavior	MASM	Modulation of hippocampal inflammation (↓TNF-α), oxidative stress (↑HO-1, ↓ROS), and autophagy (↑LC3-II, ↓p62)	*In* *vitro and* *in vivo*	Li et al. (2025)
Hepatocellular carcinoma	WM130 (C30N4H40SO5F)	↓p-EGFR, ↓p-ERK, ↓p-AKT, ↓MMP-2, ↓Bcl-2/Bax; ↑PTEN.	*In* *vitro and* *in vivo*	Qian et al. (2015)
Hepatocellular carcinoma	WM130	Suppression of stemness genes, promotion of hepatocyte markers; inhibition of AKT/GSK3β/β-catenin pathway	*In* *vitro and* *in* *vivo*	Ni et al. (2016)
Liver fibrosis	WM130	↓α-SMA; ↑PTEN; targeting of cofilin 1	*In* *vitro*	Xu et al. (2022)
Nasopharyngeal carcinoma	YYJ18 (14-Thienyl methylene matrine)	Modulation of MAPK (↓p-p38, ↑p-ERK1/2) and PI3K/Akt (↑p-Akt) pathways; activation of bax, caspase-3; inactivation of Bcl-2	*In* *vitro*	Xie et al. (2014)
Liver fibrosis	MD-1 (matrine derivatives-1)	Binding and inhibition of EGFR, ↓p-EGFR and p-Akt, ↓cyclin D1, inhibition of HSC activation	*In* *vitro and* *in vivo*	Feng et al. (2016)
Hepatocellular carcinoma	WM-127	Suppression of survivin/β-catenin pathway and expression of bax	*In* *vitro and* *in vivo*	Yin et al. (2018)
Hepatocellular carcinoma	ZS17	Activation of ROS-JNK-p53 signaling pathway; promotion of JNK phosphorylation, p53 activation, caspase cascade	*In* *vitro and* *in vivo*	Wang et al. (2022)
Gastric cancer	Compound B6 (dual inhibitor)	Dual TOPOI and PARP-1 inhibitor; induction of DNA damage, G0/G1 arrest	*In* *vitro and* *in vivo*	Qiu et al. (2024)
Lung cancer	Compound 11day (TOP1 inhibitor)	Acting as a topoisomerase I inhibitor; ↑ROS, ↑p53 and bax, ↓Bcl-2, activates caspases	*In* *vitro*	Shen et al. (2025)

### 4.3 Modern pharmacology of matrine: multi-dimensional pharmacological spectrum

Research shows that the mechanism of action of matrine is diverse and has a wide range of activities. In terms of anti-inflammatory, it can reduce the production and release of inflammatory mediators. In its anti-tumor action, matrine has been reported to inhibit tumor cell proliferation, induce apoptosis, and modulate antitumor immunity. Additionally, matrine exhibits antiviral activity, including inhibition of viral replication and infection. The janus kinase/signal transducer and activator of transcription (JAK/STAT) pathway mediates signals in immune and tumor growth. In K562 leukemia cells, matrine can inhibit JAK2/STAT3 phosphorylation, thereby reducing the expression of downstream anti-apoptotic proteins such as Bcl-xL. In a rheumatoid arthritis model, it inhibits the activation of this pathway, suppresses the proliferation of fibroblast-like synoviocytes (FLS), and induces their apoptosis (Lin et al., 2022). Additionally, Fischer’s research demonstrated that matrine and oxymatrine have no potential for gene mutation, providing key data for their safety assessment in food and medicine and pointing the direction for subsequent safety studies of the extracts (Fischer et al., 2024).

### 4.4 Pharmacokinetics of matrine: dynamics and fate

The analysis method has developed from high-performance liquid chromatography to liquid chromatography-mass spectrometry combined technology, which provides a reliable means for the determination of matrine in biological matrix, and also promotes the pharmacokinetic research of different species and matrix (Fan et al., 2013; Yang et al., 2010). Early research mainly focused on establishing a method for quantitative determination of the concentration. For example, Wu et al. first reported using HPLC to determine the concentration of matrine in rat plasma and estimate the fraction of its circulation. Wang et al. established a HPLC method for the determination of matrine in human serum, and preliminarily characterized the characteristics of the double-ventricular model of matrine in the human body after intravenous injection (Wang et al., 1994; Wu et al., 2003). With the progress of technology, higher-sensitivity LC-MS/MS and UPLC-MS/MS methods have been developed. These methods can simultaneously determine matrine and its active metabolite, thus promoting the pharmacokinetic research in dogs, rats and humans (Wang et al., 2005; Sujun Wang et al., 2005; Wu et al., 2006; Zheng et al., 2009).

Pharmacokinetic research has identified two core bottlenecks in the clinical application of matrine: low oral bioavailability and short half-life. In order to solve the problem of low bioavailability, researchers have developed a new drug delivery system. For example, Ruan et al. reported on the self-nanoemulsification delivery system (PLC-SNEDDS) based on phospholipid complexes. The system increases the absolute bioavailability of matrine from 25% to 84.6%, and the relative bioavailability reaches 338% (Ruan et al., 2010). Compared with traditional solutions or ordinary liposomes, invisible matrine lipids can extend the half-life, increase the area under the plasma concentration-time curve, and change the tissue distribution characteristics (Wu et al., 2009). Recent studies have adopted more complex models. Dr. Yan’s team established a physiological pharmacokinetic model in pigs. The model can predict the dynamic changes of matrine in the intestinal cavity and provide a basis for optimizing the administration scheme (such as 70 mg/kg every 8 h) to maintain the effective exposure level (Yang et al., 2025). In the early work, Wang and his colleagues used the classic pharmacokinetics-pharmacodynamics (PK-PD) modeling method to find that the effect and concentration relationship between matrine and oxidized matrine in rabbits is consistent with the Sigmoid Emax model (Wang and Huang, 1992; Zheng et al., 2009).

These studies show a clear development trajectory: from establishing basic analysis methods to clarifying pharmacokinetic characteristics, to developing advanced drug delivery strategies to overcome the problems of low oral bioavailability and short half-life. Eventually, complex models such as physiological pharmacokinetics are also used for accurate prediction and treatment optimization.

### 4.5 Future research directions of matrine: emerging perspectives in translational medicine

However, the current research landscape remains incomplete. Most studies focus on single pathways and lack an integrated analysis of matrine’s multi-target network and ferroptosis regulation. Although existing extraction processes have made progress, the stability of large-scale production still needs optimization. In the future, metabolomics and proteomics technologies should be combined to systematically analyze the mechanism of action of matrine. Meanwhile, a green and efficient integrated extraction-purification process should be developed to promote the clinical application of matrine. Therefore, conducting in-depth research on the chemical structure and extraction process of matrine, as well as the relationship between its traditional pharmacological effects and the ferroptosis mechanism, is of great significance for developing the applications of matrine and its derivatives in modern medicine.

### 4.6 Preclinical research of matrine: a bridge to clinical translation

As shown in Table 2, exploration has unfolded sequentially over these years. ChiCTR gradually expanded to include clinical trials assessing the therapeutic potential of matrine across conditions including postpartum vaginal microecological imbalance, rectal carcinoma, dermatitis, eczema, cancer cachexia, and refractory rheumatoid arthritis, as well as studies focused on pharmacogenomics and post-market safety. Some studies have reported preliminary results but further investigation of mechanisms and treatment regimens is still needed. Key limitations include incomplete clinical evidence, small sample sizes, and short follow-up durations. Accordingly, conduct rigorous multicenter, large-sample clinical trials to verify efficacy and safety. Systematic research on the relationship between dose and reaction is still limited, and the best treatment window is not clear. Considering individual differences, future research should be stratified according to genetic background and disease status, so as to improve the accuracy of treatment.

**TABLE 2:** Representative Clinical Trials Targeting Matrine.

Year	Registration number	Purpose of the research	Type of study	Phase	Status
2015	ChiCTR-IOC-15006913	Effect of gene polymorphism of the main components of the matrine preparation in the human body	Interventional study	N/A	Recruiting
2017	ChiCTR-IOR-17012581	Study on the therapeutic effect of sophora flavescens alkaloid gels in the treatment of aerobic vaginitis combined with bacterial vaginosis	Interventional study	N/A	Not yet recruiting
2017	ChiCTR-OPC-17012609	Clinical observation on intervention of cancer cachexia syndrome by approved traditional Chinese medicine (matrine and Kanglaite)	Observational study	Phase4	Recruiting
2018	NCT02239237	Monitor the safety of compound Kuh-seng injection	Interventional study	N/A	Recruiting
2023	ChiCTR2300070496	Effect of kushen gel therapy on genitourinary syndrome of menopause	Interventional study	N/A	Not yet recruiting
2023	ChiCTR2300071401	Effects of Kurarinone in the treatment of irritable bowel syndrome	Interventional study	Phase4	Not yet recruiting
2024	ChiCTR2400086257	Effectiveness of Kunlishu ® kushen gel in the treatment of vulvovaginal candidiasis (VVC)-multicenter, prospective, randomized, double-blind, placebo-controlled study	Interventional study	Phase4	Not yet recruiting
2024	ChiCTR2400088375	Retrospective study on the long-term efficacy and safety of Five-Flavor sophora flavescens enteric-coated capsules in the treatment of ulcerative colitis	Observational study	Phase4	Recruiting
2025	ChiCTR2500102388	Clinical comprehensive evaluation study of matrine film agent in the treatment postpartum vaginal microecological disorders	Interventional study	N/A	Recruiting

Data source: Information was obtained from the Clinical Trial Registry of the National Library of Medicine (https://clinicaltrials.gov) and Chinese Clinical Trial Registry (https://www.chictr.org.cn). Each trial was accessed and verified by using a unique NCT or ChiCTR number for each trial. The status of each trial is reported at the date of the most recent verification available in the registry.

## 5 Protective effect of matrine on the heart: from basic research to clinical translation

Matrine is currently being studied as a potential treatment for cardiovascular diseases. However, more research is still needed to clarify its optimal administration scheme, potential drug interactions, and the degree of multipath heart protection in large scale clinical trials.

### 5.1 Matrine in the treatment of sepsis-related myocardial injury

Sepsis-induced myocardial injury (SIMI) is a serious complication of sepsis, which brings a heavy burden to global health. The pathological mechanism of the disease is complex, and the existing treatment strategies have limited effect. Against this background, the natural metabolite of matrine has been found to have the potential to treat SIMI.

#### 5.1.1 Sepsis-related myocardial injury: severe challenges and treatment dilemmas

Sepsis is a major challenge in the field of global health. It is also one of the main causes of death in high-income countries. Diagnosis and treatment are constantly improving, but the incidence of sepsis continues to rise, which puts a heavy burden on the medical system (Fleischmann-Struzek et al., 2020; Markwart et al., 2020). Among the complications of sepsis, severe acute myocardial infarction (SIMI) is particularly destructive, with a mortality rate of 70%–90% (Fowler et al., 2019). The current treatment mainly relies on hemodynamic support, including individualized fluid resuscitation and the use of vasoactive drugs (Vincent, 2022). The pathological mechanism of SIMI is very complex. It involves oxidative stress, inflammatory response and cell death (Esposito et al., 2017). Identifying key mechanistic drivers and developing novel therapeutic targets remain urgent research priorities.

#### 5.1.2 Ferroptosis: a new focus in cardiovascular diseases

In recent years, the role of ferroptosis in cardiovascular diseases, including sepsis-related myocardial injury, has gradually drawn attention (Fang et al., 2023). For example, Bulluck et al. observed an association between myocardial iron deposition and adverse left ventricular remodeling in a retrospective cohort of patients with ST-segment elevation myocardial infarction, suggesting that targeting myocardial iron homeostasis may represent a novel approach to improve clinical outcomes. However, that study did not establish a causal relationship nor measure ferroptosis markers (Bulluck et al., 2016). The isolated perfused adult mouse heart model by Baba and colleagues showed that mTOR protects the heart against ferroptosis, but this *ex vivo* model lacks neurohumoral regulation, and the sensitivity of mouse myocardium to iron overload may differ from that of humans. Therefore, these studies suggest a possible value of ferroptosis in the generation of myocardial injury, but they cannot serve as direct evidence for targeting human myocardial ferroptosis with matrine (Baba et al., 2018).

Treatment with ferroptosis-specific inhibitor ferrostatin-1 (Fer-1) or iron chelating agent dextrorezoson (DXZ) can chealize free iron and inhibit lipid peroxidation. This treatment can protect mitochondrial function while reducing cardiac injury and mortality induced by amoxycillin (DOX). This protective effect is relatively specific, because apoptosis inhibitors, necrotic apoptosis inhibitors and autophagy inhibitors have failed to improve the prognosis of mice (Fang et al., 2019). Park et al. conducted proteomic analysis of cardiac tissue from mice after myocardial infarction induced by left anterior descending artery ligation. The results showed that GPX4, a key regulator of ferroptosis in cardiomyocytes, was characteristically downregulated in the early and middle stages of myocardial infarction (Park et al., 2019). In addition, antioxidant regulation and epithelial-mesenchymal transition (EMT) have been found to participate in ferroptosis regulation (Wu et al., 2021). Nrf2, the primary transcription factor regulating antioxidant responses, can prevent ferroptosis in various cell types by protecting cells from lethal ROS stress. EMT can modulate susceptibility to ferroptosis through the ZEB1-mediated adipogenic reprogramming pathway.

The Li team extended this mechanism to metabolic diseases and reported that ferroptosis plays an important role in diabetic myocardial ischemia/reperfusion injury. By inhibiting ferroptosis, endoplasmic reticulum stress (ERS) could be relieved and myocardial injury reduced. Fer-1 also alleviated injury in H9c2 cells under high-glucose conditions and during hypoxia/reoxygenation (Li et al., 2020). Zhao and colleagues found that the iron chelator deferoxamine reduced ROS levels and mitigated reperfusion injury in a rabbit heart perfusion model. Fer-1 alleviates myocardial ischemia-reperfusion injury (MIRI) in mice by blocking the lipid peroxidation chain reaction (Zhao et al., 2021). The mitochondrial iron chelator Mito-FerroGreen may mitigate doxorubicin-induced cardiomyocyte injury (Fang et al., 2023). Bi et al. reported that drugs targeting ACSL4 reduced cardiac hypertrophy in mice through the ACSL4–ferroptosis–pyroptosis pathway and that combined ferroptosis and pyroptosis inhibition improved cardiac function, suggesting a potential therapeutic approach for heart failure (Bi et al., 2024). Collectively, these findings support ferroptosis as a shared pathway of cardiac injury across diseases and suggest that ferroptosis inhibition may represent a therapeutic strategy for cardiovascular disease.

#### 5.1.3 Matrine: potential therapeutic relevance for sepsis treatment

The potential of matrine in sepsis treatment has been explored by researchers, with preliminary evidence supporting potential benefit. Liu reported that the PTENP1/MIR-106B-5P axis regulates matrine-enhanced viability of cardiac myocytes and protects cardiac myocytes from damage in sepsis (Liu et al., 2021). Xu and colleagues revealed that matrine suppresses activation of the NLRP3 inflammasome in sepsis by modulating the PTPN2/JNK/SREBP2 pathway, preventing excessive inflammatory responses (Xu Wang et al., 2023). In animal experiments, He et al. discovered that its derivative, oxymatrine (OMT), inhibited HMGB1-mediated RAGE/NF-κB activation, improved inflammation and organ damage caused by sepsis, and prolonged survival in septic mice (He et al., 2024).

#### 5.1.4 SIMI alleviation via ferroptosis inhibition with matrine

**FIGURE 2:**
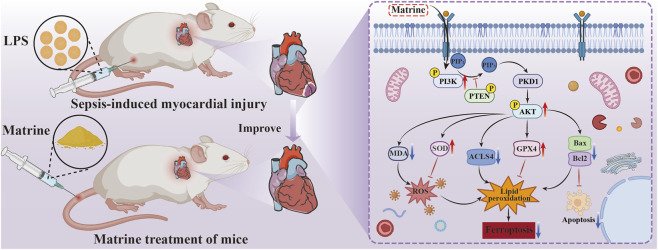
Matrine protects the septic heart by dual blockade of ferroptosis and apoptosis through selective PI3K/AKT activation (↓ROS, ↑GPX4, ↓Bax/Bcl-2), yielding higher LVEF/LVFS and less inflammation. This figure was created with BioRender.com.

The finding that matrine suppresses ferroptosis and apoptosis, thereby preventing sepsis-induced myocardial injury, suggests a potential strategy for SIMI management. Xiao used network pharmacology to predict that matrine targets relevant to SIMI are associated with ferroptosis, apoptosis, and the PI3K/AKT pathway. In an animal model of sepsis-induced myocardial injury, a low dose of 25 mg/kg matrine was superior to 50 mg/kg, improving cardiac function as evidenced by increased LVEF and LVFS, reducing inflammatory cell infiltration, lowering apoptosis, and decreasing ROS and MDA levels. Meanwhile, matrine upregulated GPX4 expression and downregulated Bax/Bcl2 and ACSL4 (Xiao et al., 2024; Xiao et al., 2023). As displayed in Figure 2, matrine alleviates sepsis-induced myocardial injury by inhibiting ferroptosis and apoptosis.

### 5.2 Matrine-mediated amelioration of atherosclerosis

Matrine has been studied as a potential treatmen for atherosclerosis (AS). It may activate the downstream antioxidant defense mechanism by targeting REG1A and restoring the PI3K/AKT/mTOR signaling pathway.

#### 5.2.1 Atherosclerosis: new breakthroughs in traditional Chinese medicine

Atherosclerosis is one of the main pathological bases of cardiovascular diseases (CVD). It is mainly characterized by lipid accumulation and inflammation in large arteries, which may ultimately lead to adverse events such as myocardial infarction (MI) and stroke (Björkegren and Lusis, 2022). In recent years, the role of ferroptosis in atherosclerosis has gradually drawn attention. These studies have shown that ferroptosis of endothelial cells may cause vascular endothelial dysfunction, thereby promoting the formation and instability of atherosclerotic plaques.

#### 5.2.2 Matrine: a potential therapeutic drug for atherosclerosis

Currently, drug therapy for atherosclerosis faces important challenges. Existing drugs not only have an unsatisfactory clinical response rate but also carry non-negligible risks of adverse effects (Fan and Watanabe, 2022; Lampsas et al., 2023; Pedro-Botet et al., 2020). Traditional Chinese medicine is increasingly being studied in this context. Previous studies have shown that natural products with multi-target mechanisms have shown encouraging efficacy profiles in preventing and treating AS and may have favorable safety profiles (Kong et al., 2025; Wang et al., 2024; Zhi et al., 2023). This suggests that screening bioactive metabolites from traditional Chinese medicine may help identify candidate agents for AS drug discovery.

#### 5.2.3 Alleviation of atherosclerosis through inhibition of endothelial cell ferroptosis by matrine

The discovery that matrine inhibits endothelial cell ferroptosis by targeting REG1A and activating the PI3K/AKT/mTOR pathway suggests potential targets and strategies for treating atherosclerosis. Zhao reported that matrine targets REG1A and activates PI3K/AKT/mTOR signaling to inhibit endothelial cell ferroptosis and alleviate atherosclerosis (Zhao et al., 2025). REG1A is a secreted protein involved in biological processes including cell survival and metabolism and may regulate cell survival and death through multiple mechanisms. REG1A expression is reduced in atherosclerosis, which may increase endothelial susceptibility to ferroptosis. Matrine increases REG1A expression, thereby enhancing endothelial antioxidant capacity and anti-ferroptotic effects. Overexpression of REG1A increases antioxidant enzyme activity, reduces lipid peroxidation, and prevents ferroptosis. The PI3K/AKT/mTOR signaling pathway regulates multiple cellular processes, including survival, proliferation, and metabolism, and its activation may reduce apoptosis and ferroptosis in endothelial cells.

**FIGURE 3:**
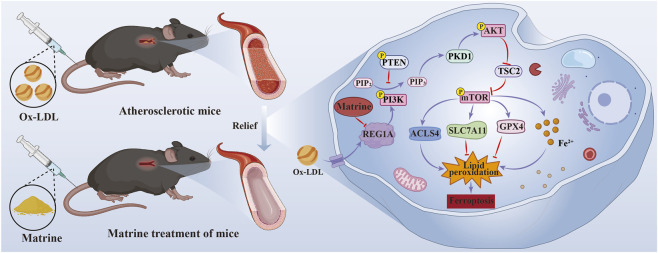
Matrine attenuates atherosclerosis by inhibiting endothelial ferroptosis: it modulates REG1A, activates PI3K/AKT/mTOR signaling, restores GPX4/SLC7A11, suppresses ACSL4, and reduces iron-driven lipid peroxidation in ox-LDL–challenged HUVECs. This figure was created with BioRender.com.

Matrine promotes activation of the PI3K/AKT/mTOR pathway, which increases antioxidant enzyme activity, decreases ROS accumulation, and prevents lipid peroxidation. Activation of PI3K/AKT/mTOR signaling also increases intracellular GSH production, improving antioxidant function and further inhibiting ferroptosis. In an atherosclerosis model, matrine binds to the REG1A protein and enhances its expression, thereby supporting PI3K/AKT/mTOR activation and downstream antioxidant gene transcription. Experiments indicate that in an HUVEC injury model induced by 100 μg/mL ox-LDL, matrine alleviates ox-LDL–induced reductions in HUVEC viability in a concentration-dependent manner. At 3 mg/mL, matrine reduced intracellular Fe^2+^ by 40% and lipid peroxidation products by 35%. Meanwhile, it upregulated GPX4 and SLC7A11, enhanced GSH synthesis capacity, and inhibited lipid peroxidation-mediated ferroptosis. *In vivo* experiments confirm that matrine inhibits ferroptosis in the aorta of ApoE−/− mice fed a high-fat diet and alleviates arterial lesions. In ApoE^-^/^-^ mice, intragastric administration of matrine at 200 mg/kg/day for 12 weeks reduced aortic lipid plaque area by 32%. Expression of ferroptosis-promoting proteins such as ACSL4 in myocardial tissue was downregulated. Meanwhile, phosphorylated AKT and mTOR levels increased by 25%–30%, suggesting that PI3K/AKT/mTOR activation is a core mechanism underlying its anti-atherosclerotic effect (Zhao et al., 2025). As displayed in Figure 3, matrine alleviates atherosclerosis by inhibiting endothelial ferroptosis through targeting REG1A and activating PI3K/AKT/mTOR signaling.

### 5.3 Preclinical evidence and translational considerations: multi-pathway regulatory properties of matrine in cardiovascular disease

The multi-pathway effects of matrine (such as inhibiting ferroptosis, activating antioxidant reactions and regulating lipid metabolism) may support its application in the treatment of cardiovascular diseases. It is reported that the structurally optimized matrine derivatives such as WM130 and MASM have binding affinity with REG1A. In animal models, the anti-atherosclerotic effect is enhanced by more than 50% and the toxicity is reduced. Preclinical studies show that the combination of matrine and traditional heart failure drugs may improve heart function. However, metabolic interactions involving CYP450 enzyme may affect the safety of such combinations. Based on the chemical structure and pharmacological effect of matrine, people have studied its therapeutic potential for cardiovascular diseases. In the sepsis-related myocardial injury and atherosclerosis model, matrine may play a protective role in the heart in a variety of ways, including inhibiting ferroptosis, activating antioxidant reactions and regulating lipid metabolism. Future research should include mechanism research, optimization of dosage and administration schemes, and multi-center, large-sample clinical trials to better clarify its efficacy and safety, and support its clinical transformation and application.

## 6 Investigation into the therapeutic potential and underlying mechanisms of matrine in experimental autoimmune encephalomyelitis

Experimental autoimmune encephalomyelitis (EAE) is widely used as the study of multiple sclerosis (MS) because it recapitulates key immune-mediated pathological features in the central nervous system (CNS) observed in MS. In recent years, ferroptosis has been implicated in the development of EAE. Emerging evidence suggests that matrine may alleviate EAE in preclinical models. In animal studies, it can reduce clinical scores, attenuate demyelination lesions and inflammatory infiltration, and modulate immune regulatory pathways. Although current research is largely limited to animal models, the reported neuroprotective effects and favorable preclinical safety profile support further translational investigation.

### 6.1 Experimental autoimmune encephalomyelitis: a simulated battlefield for multiple sclerosis

Experimental autoimmune encephalomyelitis is a well-established model for studying the pathogenesis and treatment of multiple sclerosis, characterized by immune-mediated inflammation and demyelination in the central nervous system. Multiple sclerosis is an autoimmune-mediated central neurodegenerative disease, characterized by abnormal detachment of the myelin sheath that surrounds axons. This damage can lead to motor impairment and cognitive decline and may patients toward progressive disability and reduced quality of life (McGinley et al., 2021). The major pathological features of MS include inflammation of the central nervous system, demyelination, and neurodegeneration. These features are highly reproducible in the EAE model, making it an ideal platform for the study of MS.

### 6.2 Ferroptosis: a new player in the pathogenesis of EAE

In recent years, ferroptosis, a novel form of regulated cell death, has been suggested to contribute to the pathogenesis of EAE (Costa et al., 2023; Luoqian et al., 2022; Tang et al., 2025; Van San et al., 2023). Ferroptosis-associated microglial activation can promote inflammation, leading to increased release of inflammatory factors such as TNF-α, IL-1β, and IL-6 in the EAE model. The release of inflammatory factors will aggravate inflammation and damage to the central nervous system. This process not only aggravates nerve cell damage, but also may further promote ferroptosis, thus forming a positive feedback loop.

### 6.3 Matrine: a new hope for MS treatment

To date, multiple studies have evaluated the effects of matrine on MS in experimental models. For instance, Zhao found that clinical scores, inflammatory cell infiltration in the CNS, and demyelinating lesions in rats treated with matrine were improved compared with the untreated group. In addition, high-dose matrine reduced serum levels of pro-inflammatory factors IL-23 and IL-17 (Zhao et al., 2011). Kan et al. indicated that matrine may exert immunomodulatory effects by inhibiting recruitment of immune cells into the CNS (Kan et al., 2015). Zhu’s experimental results suggested that matrine may improve the course of EAE by reducing oligodendrocyte (OLG) apoptosis through restoration of the proNGF:NGF balance and the ratio of its receptors p75(NTR):TrkA (Zhu et al., 2016). Zhao reported that matrine regulates the inflammatory IL-33/ST2 axis (Zhao et al., 2016). Zhang et al. found that matrine can induce glial cells of the CNS to secrete neurotrophin 3, which may help protect nerve cells from inflammation-associated injury (Zhang et al., 2017).

Fan found that the matrine derivative (6aS, 10S, 11aR, 11bR, 11 cS) −10 -methylaminododecahydro - 3a, 7a - diazabenzo (de)anthracene- 8 - thione (MASM) significantly alleviated EAE progression (Fan et al., 2022). Ma’s team found that matrine promoted maturation of oligodendrocytes and myelin repair by regulating the Wnt/β-catenin/TCF7L2 signaling pathway (Ma et al., 2022). Chhabra reported that matrine improved multiple sclerosis-related outcomes, and the underlying mechanism may be associated with regulation of the STAT-3/mTOR/PPAR-gamma signaling pathway (Chhabra et al., 2023). Dou et al. found that matrine treatment could regulate the gut microbiota and metabolites, thereby delaying multiple sclerosis progression (Dou et al., 2024). Collectively, these studies indicate that matrine and its derivatives may exert anti-MS/EAE effects through multiple mechanisms, including immune modulation, glial protection, regulation of key signaling pathways, and potential involvement of the gut–brain axis.

### 6.4 Matrine alleviating pathological damage in EAE by inhibiting the ferroptosis pathway

Interestingly, Feng and his colleagues found that in experimental autoimmune encephalomyelitis, matrine could significantly alleviate neuroinflammation and demyelination by regulating iron metabolism and redox balance. When model mice were intraperitoneally injected with 50 mg/kg/d matrine, the clinical neurological function score decreased by 40%, the level of lipid peroxidation product MDA in spinal cord tissue dropped by 35%, and the activities of antioxidant molecules GSH and SOD increased by 28% and 32%, respectively. Meanwhile, the expression of key ferroptosis proteins GPX4 and SLC7A11 was upregulated by 25%–30%, and the expression of pro-ferroptosis factors LPCAT3 and PTGS2 was downregulated by 40%–50%. Immunofluorescence results showed that matrine significantly reduced the co-expression of pro-inflammatory factors IL-6, TNF-α, and ferroptosis-related enzymes LOX, PTGS2 in microglia (IBA-1+), and inhibited their polarization towards the pro-inflammatory phenotype (Feng et al., 2024).

**FIGURE 4:**
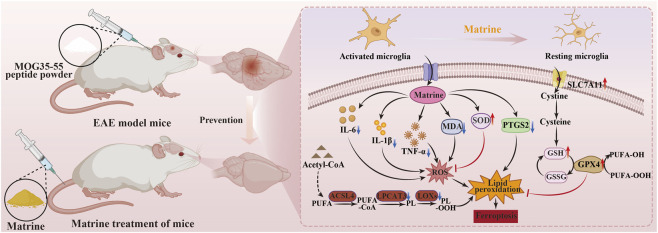
Matrine ameliorates EAE by modulating microglial ferroptosis: it restores the SLC7A11-GPX4 antioxidant axis (↑GSH/SOD, ↓MDA/LOX/PTGS2), reduces the pro-ferroptosis/pro-inflammatory phenotype (↓IL-6, TNF-α, IL-1β), and thereby decreases CNS lipid peroxidation, demyelination and clinical deficits. This figure was created with BioRender.com.

Mechanistically, matrine exerts its effects through a dual pathway. On one hand, it activates the SLC7A11/GPX4 antioxidant axis to enhance glutathione synthesis and the ability to scavenge lipid peroxides. On the other hand, it inhibits NCOA4-mediated ferritinophagy, thereby reducing the release of free iron and blocking the iron-dependent lipid peroxidation chain reaction. In addition, matrine downregulates the activity of the TLR4/NF-κB pathway in microglia, which in turn reduces the secretion of pro-inflammatory mediators such as IL-1β and IFN-γ, forming an “anti-ferroptosis - anti-inflammation” synergistic effect. In the spinal cord injury area, the density of microglia in the matrine treatment group was 22% lower than that in the model group. Moreover, the proportion of M1-type pro-inflammatory microglia decreased from 68% to 34%, while the proportion of M2-type anti-inflammatory phenotypes increased accordingly (Feng et al., 2024). As displayed in Figure 4, matrine prevents experimental autoimmune encephalomyelitis through regulating the detailed mechanisms of ferroptosis in microglia.

### 6.5 Preclinical evidence and translational considerations: future prospects of matrine

At the clinical transformation level, matrine may provide a treatment strategy for autoimmune diseases of the central nervous system. At present, the research is still limited to animal models. The reported neuroprotective effects (such as improving motor function and delaying the progression of demyelination in the EAE model) and good preclinical safety have laid the foundation for follow-up research. In the future, researchers need to further evaluate its blood-brain barrier penetration efficiency and examine the safety of long-term use.

## 7 Protective effect of matrine on lung tissue: defense mechanism from severe acute pancreatitis to multiple organ injury

Severe acute pancreatitis (SAP) is an inflammatory disease. It can progress to systemic injury. Acute lung injury (ALI) is the main complication of SAP and an important factor leading to early death. At present, there are more and more studies exploring the relationship between ferroptosis and SAP-related ALI. Against this background, matrine is also studied. Preclinical evidence shows that matrine may have a protective effect on lung damage.

### 7.1 Severe acute pancreatitis: systemic inflammatory response and lung injury

Severe acute pancreatitis is a serious inflammatory disease. The disease can quickly spread beyond the pancreas, causing systemic inflammatory response syndrome (SIRS) and multiple organ dysfunction syndrome (MODS) (Liu et al., 2022). The lungs are one of the most often affected organs. In SAP, lung tissue usually shows increased inflammation, excessive oxidative stress and cell death. These changes can lead to acute respiratory distress syndrome (ARDS). ARDS is one of the main causes of early death in SAP.

### 7.2 Ferroptosis: a new pathological mechanism of pulmonary diseases

In recent years, research into the relationship between ferroptosis and acute lung injury after severe acute pancreatitis has increased. For instance, Ge and colleagues performed network pharmacology, metabolomics, and 16S rDNA sequencing to identify key mechanisms by which Qingyi Decoction may modulate ferroptosis in lung tissue during SAP-ALI in a rat model (Ge et al., 2023). The Yang team investigated cell therapy and reported that mesenchymal stromal cells (MSCs) may have therapeutic potential for SAP-ALI (Yang et al., 2024). Shen reported that emodin (EMO) can inhibit ferroptosis both *in vivo* and *in vitro*. In addition, emodin exerts protective effects by regulating the Nrf2/HO-1/GPX4 signaling pathway, suggesting a potential mechanism relevant to SAP-ALI (Shen et al., 2025). Collectively, these studies indicate that ferroptosis plays an important role in the occurrence and development of pulmonary diseases.

### 7.3 Matrine: a potential therapeutic drug for lung diseases

The potential of matrine in the treatment of lung diseases has attracted attention. Many studies have shown that its mechanism of action is multifaceted. Zhao et al. reported that matrine protects the lungs by regulating macrophage polarization and inhibiting cell apoptosis (Zhao et al., 2021). The Luo team found that matrine derivative Sophflarine A can inhibit the proliferation of NSCLC cells through the PI3K/AKT/mTOR pathway (Luo et al., 2023b). Wang confirmed that matrine inhibits the activation of NLRP3 inflammatory corpuscles in sepsis by regulating the PTPN2/JNK/SREBP2 pathway (Xu Wang et al., 2023). Another study shows that matrine can overcome the chemotherapy resistance of NSCLC by regulating the DNA repair mechanism (Wang et al., 2024). Zhao also found that matrine relieves acute pneumonia caused by *Staphylococcus aureus* by blocking the inflammatory signaling pathway mediated by MLKL/NLRP3 (Zhao et al., 2025b).

### 7.4 Matrine suppressing ferroptosis in acute lung injury caused by severe acute pancreatitis

Jin et al. reported that matrine can reduce inflammation, oxidative damage, and ferroptosis in lung tissue in SAP. This shows that it has a protective effect on SAP-ALI. The mechanism may involve the activation of the mitochondrial antioxidant pathway UCP2/SIRT3/PGC1α in lung tissue (Jin et al., 2023). UCP2, SIRT3, and PGC1α are key regulators of cellular redox balance and metabolism. UCP2-mediated uncoupling can reduce ROS generation within mitochondria. SIRT3, an NAD + -dependent mitochondrial deacetylase, regulates multiple antioxidant enzyme activities. PGC1α is a transcriptional coactivator that promotes mitochondrial biogenesis and increases antioxidant enzyme expression. Activation of the UCP2/SIRT3/PGC1α pathway during SAP may alleviate oxidative stress and ferroptosis by inducing UCP2, promoting proton leak, suppressing ROS production, activating SIRT3 and its downstream coactivator PGC1α, enhancing mitochondrial biogenesis, and increasing expression of antioxidant enzymes including GPX4, HO-1, and NQO1, thereby inhibiting ferroptosis in SAP-associated ALI (Jin et al., 2023). Experiments showed that pretreatment with 200 mg/kg matrine reduced the pathological score of lung tissue in SAP mice by 40%, myeloperoxidase (MPO) activity by 58%, and serum IL-6 and TNF-α levels by 62% and 55%, respectively. It also reversed increases in MDA and depletion of GSH in lung tissue (Jin et al., 2023).

**FIGURE 5:**
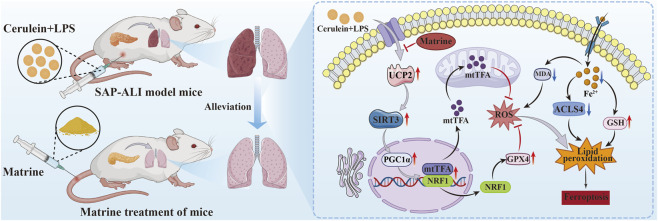
Matrine protects lungs from SAP-ALI by activating the UCP2-SIRT3-PGC1α axis: it reduces mitochondrial ROS generation, increases HO-1/NQO1/GPX4, lowers ACSL4-Fe^2+^-MDA, and thereby reduces ferroptosis-driven oxidative lung damage. This figure was created with BioRender.com.

*In vitro* studies indicated that in BEAS-2B and MLE-12 lung epithelial cells, 1 mg/mL matrine could inhibit the LPS-induced accumulation of Fe^2+^ and the production of lipoxygenase product 12(S)-HETE. However, siRNA-mediated knockdown of UCP2 completely counteracted these effects, causing the cell mortality rate to rebound from 28% to 65%. Overexpression of UCP2 reduced the intracellular ROS level by 73% compared with the LPS group by enhancing the activity of the SIRT3/PGC1α pathway, which verified the mechanism by which matrine alleviates inflammation, oxidative stress, and excessive ferroptosis in lung tissue during SAP via the UCP2/SIRT3/PGC1α pathway (Jin et al., 2023). As displayed in Figure 5, matrine reduces acute lung injury induced by severe acute pancreatitis through alleviating ferroptosis.

### 7.5 Preclinical evidence and translational considerations: future application prospects of matrine

The mechanism through which matrine activates the mitochondrial antioxidant system via the UCP2/SIRT3/PGC1α pathway offers a new direction for developing natural drugs targeting acute lung injury. Future studies may further investigate the optimization of the dose window of matrine, methods of administration, and synergy with other supportive treatments, such as mechanical ventilation, to enhance the feasibility and safety of its clinical application. The combination of matrine with antioxidants or anti-inflammatory drugs may also enhance its protective effect. For example, in combination with NAC or other antioxidants, it may be more effective to reduce severe acute lung injury. Direct clinical evidence supporting matrine’s efficacy via ferroptosis modulation remains lacking. Overinterpretation of preclinical data as clinical readiness is cautioned.

## 8 Effects of matrine on cervical cancer: ferroptosis mechanism and clinical translation potential

Cervical cancer brings a heavy health burden to the world, resulting in hundreds of thousands of deaths every year. Ferroptosis has attracted attention in recent years as a potential tumor treatment mechanism, and matrine has therefore been studied as a candidate metabolite in this field.

### 8.1 Cervical cancer: a major threat to Women’s health

Cervical cancer accounts for approximately 6.5% of global female cancer cases (Jha et al., 2023). In 2020, global estimates included 604,127 new cases and 341,831 deaths (Barnholtz-Sloan et al., 2009; Howe et al., 2006; Sherman et al., 2005). Standard therapies such as surgery, radiotherapy and chemotherapy can control the progression of some patients’ condition. However, drug resistance and recurrence are still the main obstacles to treatment.

### 8.2 Ferroptosis: a new dawn in cancer treatment

In recent years, ferroptosis, a novel form of cell death, has drawn considerable attention for its potential in cancer treatment (Gao et al., 2022). This trend is also reflected in cervical cancer research. For instance, Luo et al. discovered that tumor-associated macrophages (TAMs) can secrete exosomes carrying miR-660–5p to inhibit the ferroptosis process in cervical cancer cells (Luo et al., 2023). In contrast, the Zhou team reported that chrysotoxine (CTX) can effectively trigger ferroptosis in cervical cancer cells (Zhou et al., 2025).

### 8.3 Matrine: a new hope for cervical cancer treatment

In recent years, studies have investigated matrine and related traditional Chinese medicine formulations in cervical cancer. Li et al. reported that Compound Kushen Injection, used as an adjuvant, showed synergistic effects in cervical cancer patients (Li et al., 2023). Compound Kushen Injection (CKI) is a standardized botanical drug preparation approved by the National Medical Products Administration (NMPA) of China. Its composition comprises two principal botanical drugs: aqueous extracts of *Sophora flavescens Aiton* (Fabaceae) roots, standardized to contain matrine and oxymatrine as marker metabolites; and extracts of Smilax glabra Roxb (Smilacaceae) rhizomes. In addition, multiple *in vitro* studies have reported that matrine derivatives may inhibit tumor cell proliferation (Wang et al., 2025).

### 8.4 Mechanisms of action of matrine: activation of piezo1 and induction of ferroptosis

Jin et al. reported that matrine induces ferroptosis in cervical cancer cells via Piezo1. The innovation of this finding lies in revealing the possibility that mechanosensitive ion channels may serve as upstream regulators of ferroptosis (Jin et al., 2024). Mechanistically, matrine triggers an influx of Ca^2+^ by promoting the expression of Piezo1 protein, thereby activating the calcium-dependent signaling cascade, leading to an increase in intracellular iron ion levels, and promoting ferroptosis. This process is independent of traditional ferroptosis regulators like xCT and transferrin receptor (Tfr) and further inhibits the activity and expression of GPX4, leading to enhanced lipid peroxidation product MDA increased by 68%, free Fe^2+^ increased by 52%, and ROS by 73%, inducing ferroptosis in cervical cancer cells (Jin et al., 2024).

**FIGURE 6:**
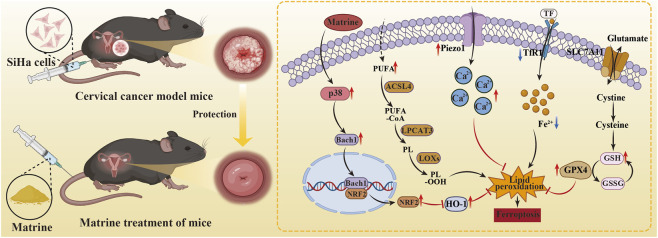
Matrine induces ferroptosis in cervical cancer by activating Piezo1-Ca^2+^ signaling: it increases free Fe^2+^ (↑TfR1, ↓ferroportin/FTL), promotes PUFA-lipid peroxidation (↓GPX4), and suppresses antioxidant responses via p38-Bach1-Nrf2 repression and PI3K/Akt/mTOR inhibition, resulting in iron-catalyzed lipid ROS accumulation. This figure was created with BioRender.com.

In vitro experiments, the researchers found that matrine inhibited the viability of SiHa cells in a dose-dependent manner, reduced the colony formation rate, and increased LDH release (Jin et al., 2024). In animal models, the mice treated with matrine had smaller tumor volumes and less serious pathological damage. These findings were further validated by the significantly increased expression levels of ferroptosis markers within the tumor tissues. For example, an intraperitoneal injection of 75 mg/kg matrine was able to shrink the tumor volume by 58% and decrease tumor weight by 63% in SiHa tumor-bearing mice. Additionally, the HE staining of liver and kidney functions presented no obvious damage in each group (Jin et al., 2024). According to the GEO dataset, the gene sets of ferroptosis-related targets, including ACSL4 and PTGS2, were significantly enriched in U937 cells treated with matrine. This indicates that such an effect is related to the ferroptosis pathway. Piezo1 siRNA transfection can completely reverse the increase of Fe^2+^, the downregulation of GPX4, and cell death induced by matrine. Besides, the Piezo1 agonist Yoda1 can promote this effect in synergy. However, several methodological limitations constrain the strength of their conclusions. Firstly, although Yoda1, a Piezo1 agonist, synergistically enhanced the effect, no Piezo1 antagonist or Piezo1 knockout cells were used for reverse validation. Secondly, the study did not employ a Ca^2+^ chelator (BAPTA-AM) or perform extracellular Ca^2+^ removal experiments, making it impossible to distinguish between Ca^2+^ influx and Ca^2+^ release from the endoplasmic reticulum. Thirdly, knockdown of xCT or Tfr by siRNA did not alter the effect, but the expression levels of xCT/SLC7A11 themselves were not detected, leaving the possibility of compensatory upregulation unresolved. As shown in Figure 6, cervical cancer cells develop active Piezo1 and subsequently undergo ferroptosis because of matrine.

### 8.5 Preclinical evidence and translational considerations: potential and challenges of matrine

The mechanism of matrine inducing ferroptosis by activating Piezo1 provides a new strategy for cervical cancer treatment in clinical translation. This can be regarded as an effective method of inhibiting the proliferation and growth of tumor cells and may overcome the problem of drug resistance in traditional methods of treatment. There is no significant change in the body weight of tumor-bearing mice, and the serum ALT and CRE levels are similar to those in the control group. It has no overlapping mechanisms with cisplatin. When combined for use, it enhances the antitumor effect, increasing the tumor volume inhibition rate to 71% without obvious additive toxicity, indicating its potential as an adjuvant therapeutic drug (Jin et al., 2024). However, there are still some challenges to be overcome. Ferroptosis might damage normal cells, and high-concentration matrine might bring toxic reactions to normal tissues. Therefore, in clinical use, the dosage and administration method of matrine should be strictly controlled to avoid unnecessary damage to normal cells. Furthermore, based on existing indirect evidence (e.g., matrine regulates the Nrf2/GPX4 axis and modulates ROS and iron metabolism), we reasonably hypothesize the potential ferroptosis relevance in other cancers and anticipate the ferroptosis-regulating potential of matrine in other malignancies, such as liver cancer, pancreatic cancer, and colorectal cancer.

## 9 The cardiotoxicity, nephrotoxicity, and hepatotoxicity of matrine: mechanism exploration and clinical translation strategies

Although matrine has demonstrated therapeutic potential, it has also been associated with cardiotoxicity, nephrotoxicity, and hepatotoxicity, which may limit its application.

### 9.1 Cardiotoxicity caused by matrine: ferroptosis and disturbances in the antioxidant system

MT-induced cardiotoxicity has been linked to ferroptosis induction and disruption of the Nrf2 antioxidant defense system. Fer-1 (a ferroptosis inhibitor) or dimethyl fumarate (an Nrf2 agonist) have been reported to increase cell survival to 83% and reduce MDA levels by 51%. Sodium selenite (SS) reduces ROS accumulation by stimulating GPX4 activity. Together, these findings suggest that Nrf2 activation or selenium supplementation may represent potential detoxification strategies. However, translation remains limited, and further *in vivo* studies are needed to define the toxic dose range of matrine and evaluate potential interactions with cardioprotective medications. In addition, formulation strategies, including structural modification or combination with antioxidants, may help improve the balance between therapeutic efficacy and cardiac toxicity.

#### 9.1.1 Mechanisms of action of matrine on cardiotoxicity

Matrine cardiotoxicity has been associated with induction of cardiomyocyte ferroptosis and impairment of the Nrf2 antioxidant system (Wang J. et al., 2024). Under physiological conditions, Nrf2 (nuclear factor E2-related factor 2) regulates intracellular redox balance by promoting expression of antioxidant genes. Matrine has been reported to inhibit Nrf2 nuclear translocation, leading to reduced expression of downstream antioxidant proteins including xCT (cystine/glutamate transporter), GPX4 (glutathione peroxidase 4), and FSP1 (ferroptosis suppressor protein 1). This may result in depletion of glutathione (GSH) and reduced antioxidant capacity in cardiomyocytes. Mechanistic studies further suggest that matrine inhibits Nrf2 transcriptional activity by reducing its binding to antioxidant response elements in the promoter regions of GPX4 and FSP1, thereby suppressing GSH-related antioxidant defenses.

#### 9.1.2 Experimental research on manifestations of cardiotoxicity and intervention

In H9c2 cardiomyocytes exposed to 1.5 mg/L MT for 24 h, cell viability decreased to 52% and lactate dehydrogenase release increased approximately threefold, indicating membrane injury. Fe^2+^ content increased by 40%, and mitochondrial membrane potential (ΔΨm) depolarized by 45%, consistent with impaired mitochondrial function. The ferroptosis inhibitor Fer-1 and the Nrf2 agonist dimethyl fumarate (DMF) were reported to reverse these changes, restoring cell survival to 83% and reducing MDA by 51%. Sodium selenite suppressed ROS accumulation and improved mitochondrial function by increasing GPX4 activity. These findings suggest that Nrf2 activation or selenium supplementation may mitigate matrine-associated cardiotoxicity, although further validation in relevant contexts is required (Wang J. et al., 2024).

#### 9.1.3 Challenges and prospects at the clinical translation level

At the clinical translation level, cardiotoxicity may limit widespread clinical use of matrine in indications such as cancer and hepatitis. However, Nrf2 activators or selenium preparations may offer approaches to manage toxicity. For example, *in vitro*, DMF decreased matrine-induced cardiomyocyte death by 40% by activating Nrf2 transcription and increasing GPX4 and FSP1 expression. CoQ10 showed synergistic protection by supporting FSP1-mediated ubiquinone reduction, decreasing lipid peroxidation and free radical accumulation (Wang J. et al., 2024). Most current investigations are based on cell models, and the translational gap remains substantial: key parameters such as the *in vivo* toxic dose range, the pharmacokinetic profile of matrine in cardiac tissue, and the potential for drug–drug interactions with cardioprotective agents remain undefined. Future studies should evaluate combined preparations of matrine with antioxidants and assess whether structural modification can reduce suppression of the Nrf2 pathway. Moreover, exploring liver-targeted delivery systems to minimize systemic exposure may be a strategy to reduce off-target cardiotoxicity while preserving therapeutic efficacy.

### 9.2 Matrine-induced nephrotoxicity: the key role of ferroptosis and the synergistic mechanism of multiple pathways

Matrine-induced nephrotoxicity may be related to the kidney’s role in drug excretion and susceptibility to oxidative injury. It has been reported to disrupt antioxidant defenses (e.g., GSH depletion and inhibition of GPX4 activity) and iron homeostasis (e.g., Fe^2+^ accumulation and upregulation of TFR1/ACSL4), thereby promoting ferroptosis and lipid peroxidation. Sodium selenite (SS) has been reported to activate the GSH–GPX4 axis in a dose-dependent manner (including upregulating GPX4 and restoring xCT/CTH), with nephroprotective effects comparable to Fer-1. However, further work is needed to define a safe and effective selenium dose window in humans and to evaluate SS–matrine combination regimens or other GPX4-targeted selenium metabolites to support renal safety during matrine treatment.

#### 9.2.1 Reasons and manifestations of the kidney’s susceptibility to matrine toxicity

As a crucial organ for drug metabolism and excretion, the kidney is susceptible to drug-induced toxicity. Recent studies suggest that high-dose matrine may cause renal toxicity, and the mechanism appears to involve induction of ferroptosis in renal cells through disruption of antioxidant defenses and iron homeostasis (Wang F. et al., 2024). In mice treated with matrine (100 mg/kg/day for 20 days), renal structure was severely damaged, with shedding of tubular epithelial cells, loss of the brush border, and glomerular fibrosis. In addition, serum blood urea nitrogen (BUN), creatinine (Scr), and kidney injury molecule Kim-1 increased, indicating impaired renal function. In NRK-52E cells treated with matrine, cell death increased, further supporting a direct toxic effect on renal cells (Wang F. et al., 2024).

#### 9.2.2 The key role of ferroptosis in nephrotoxicity and the multi-pathway synergistic mechanism

Matrine increased levels of free Fe^2+^, reactive oxygen species, and lipid peroxidation products in renal tissues while decreasing GSH content and GPX4 activity. It also upregulated expression of TFR1 and acyl-CoA synthetase 4, consistent with iron-dependent lipid peroxidation as a key mechanism of renal injury. *In vitro*, NRK-52E cells treated with 1 mg/mL matrine showed decreased viability and increased LDH release. The ferroptosis inhibitor Fer-1 reversed these changes, supporting the role of ferroptosis in matrine-induced nephrotoxicity (Wang F. et al., 2024). In addition, matrine may reduce cysteine availability and GSH synthesis by inhibiting xCT/CTH-mediated pathways and may promote iron influx by activating TFR1, which can further enhance oxidative stress (Wang F. et al., 2024). Matrine may also exacerbate mitochondrial dysfunction by reducing ΔΨm, potentially amplifying cell injury.

#### 9.2.3 Protective effect and clinical translation potential of sodium selenite

Recent research suggests that SS can inhibit ferroptosis by activating the GSH–GPX4 antioxidant axis, thereby alleviating matrine-induced nephrotoxicity. In NRK-52E cells, pretreatment with 0.five to one μM SS upregulated GPX4 expression and activity in a dose-dependent manner and restored levels of xCT and cystathionine γ-lyase (CTH) that were inhibited by matrine, promoting cysteine production and GSH synthesis. Mechanistic studies showed that SS reversed matrine-induced decreases in GPX4, CTH, and xCT protein levels. When the GPX4 inhibitor RSL3 was used, or when GPX4, CTH, or xCT was knocked down by siRNA, the protective effect of SS was eliminated, suggesting that SS acts through the GSH–GPX4 system. *In vivo*, administration of 100 μg/kg SS in combination with matrine reduced Fe^2+^ and lipid peroxidation in renal tissues, restored GSH/GPX4 function, and improved renal pathological scores (Wang F. et al., 2024).

At the clinical translation level, sodium selenite, a low-toxicity selenium preparation, exhibits a renal protective effect comparable to that of ferroptosis inhibitors, without obvious toxicity superposition. Its mechanism of enhancing GPX4 activity by supplementing selenium provides a new strategy for toxicity management in the clinical application of matrine. However, it is necessary to further verify the pharmacokinetic characteristics and long-term safety of SS in humans, especially the precise regulation of the selenium dose window, to avoid the potential risk of selenium poisoning. The current evidence is confined to a single dose and duration of matrine-induced injury; future studies must delineate the dose-time-effect relationship to define the optimal intervention window for SS. In addition, combination therapy with SS and matrine or synthesis of new selenium metabolites targeting GPX4 may be studied to further improve the renal safety of anti-tumor therapy and provide safer guarantees for the clinical application of matrine.

### 9.3 Matrine-induced hepatotoxicity: a vicious cycle of ferroptosis and the inhibition of the antioxidant system

Matrine-associated hepatotoxicity has been linked to disruption of the Nrf2/GPX4 antioxidant axis and induction of ferroptosis. The ferroptosis inhibitor Fer-1 and the Nrf2 agonist t-BHQ have been reported to increase cell survival to 83%, and selenium preparations may alleviate ferroptosis-associated injury by activating GPX4. These findings suggest that targeting this pathway may help mitigate toxicity. Future work should evaluate low-dose matrine–antioxidant regimens, liver-targeted delivery systems to reduce exposure, and biomarkers of the Nrf2/GPX4 pathway for early toxicity monitoring.

#### 9.3.1 Mechanisms of action of matrine on hepatocyte toxicity

Hepatocytes are the main functional cells of the liver, and normal liver function depends on their viability. Matrine has been reported to induce ferroptosis by inhibiting the Nrf2/GPX4 antioxidant system, leading to reduced expression of proteins such as xCT and GPX4, depletion of hepatocyte GSH, increased lipid peroxidation products, and accumulation of free iron (Fe^2+^). Matrine may inhibit Nrf2 nuclear translocation and binding to antioxidant response elements (AREs), thereby downregulating key downstream proteins including xCT/SLC7A11, GPX4, HO-1, and FSP1. Dual-luciferase reporter assays suggested that matrine reduces Nrf2 binding to ARE sites at −580/−590 bp and −503/−513 bp of the GPX4 promoter and at −1691/−1701 bp of the xCT promoter (Wang et al., 2023). Reduced expression of these proteins may decrease GSH synthesis and GPX4 activity, limiting lipid peroxide detoxification and thereby promoting iron-dependent lipid peroxidation damage. In addition, matrine may promote iron influx by upregulating TFR1 and DMT1 while inhibiting FTH, causing accumulation of free iron (Fe^2+^). Together, these effects can amplify oxidative stress and create a feed-forward loop between antioxidant system suppression, iron overload, and lipid peroxidation (Wang et al., 2023b).

#### 9.3.2 Experimental research and intervention strategies

In a BALB/c mouse model, continuous intraperitoneal injection of 75–100 mg/kg matrine for 15 days caused liver injury, including an 18%–25% increase in liver weight, elevations of serum ALT and AST by 2.3-fold and 1.9-fold, and histopathological changes such as inflammatory cell infiltration, hepatocyte swelling, and disruption of hepatic plate structure. *In vitro*, treatment of L02 hepatocytes with 1.5 mg/mL matrine for 24 h reduced cell viability to 52%, increased ROS by 68%, and depolarized ΔΨm by 45% (Wang et al., 2023). The ferroptosis inhibitor Fer-1 and the Nrf2 agonist t-BHQ reversed these changes and increased cell survival to 83%. Selenium supplementation also reduced matrine-induced MDA elevation and Fe^2+^ accumulation by enhancing GPX4 activity, suggesting that Nrf2 activation or selenium supplementation may inhibit ferroptosis and mitigate hepatotoxicity (Wang et al., 2023).

#### 9.3.3 Clinical application prospects and challenges

**FIGURE 7:**
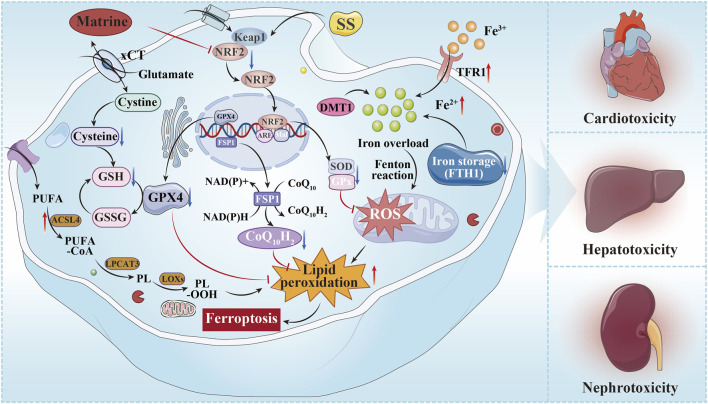
Matrine induces lipid peroxidation and ferroptosis by inhibiting Nrf2 nuclear translocation and downregulating the expression of its downstream antioxidant proteins, including xCT and GPX4, as well as disrupting iron homeostasis, resulting in intracellular Fe^2+^ accumulation. This mechanism represents the primary pathway underlying matrine-induced multi-organ toxicity in the heart, liver, and kidney. This figure was created with BioRender.com.

In terms of clinical application prospects, hepatotoxicity restricts the wide use of matrine in anti-tumor and anti-inflammatory treatments in clinical settings. Recent evidence indicates that combination regimens incorporating Nrf2 agonists or selenium-based metabolites could lessen matrine-induced hepatotoxicity. However, caution is warranted regarding potential pharmacodynamic antagonism, as Nrf2 activation might theoretically interfere with the pro-oxidant mechanisms underlying matrine’s anticancer activity. In the coming years, optimized medication regimens, such as low-dose matrine together with antioxidants or targeted delivery systems that can avoid exposure to the liver, need to be explored. Meanwhile, biomarkers for the Nrf2/GPX4 system can serve as an early predictor for hepatotoxicity risk. Mechanism-driven research is required for the development of safe medication strategies. As mentioned earlier, matrine is a traditional Chinese medicinal substance with significant diversity in its pharmacological activities. Its own cardio-toxicity, nephrotoxicity, and hepatotoxicity display high complexities. The research on its mechanisms of toxicity from the induction of ferroptosis to interference with the antioxidant system, from single-pathway modulation to multi-pathway synergy action, could offer novel perspectives for the management of its toxicities in clinics. No study has evaluated matrine administration beyond 30 days in any animal model. Chronic ferroptosis modulation—whether inhibition or induction—carries theoretical risks. Prolonged suppression of ferroptosis via Nrf2/GPX4 activation might delay physiological clearance of damaged cells, potentially facilitating malignant transformation or fibrosis. Conversely, sustained induction of ferroptosis could affect iron-rich organs. Future studies must include at least 3–6months toxicology studies, with histopathological examination of all major organs and assessment of reproductive, developmental, and carcinogenic potential. As displayed in Figure 7, matrine is currently explored in the mechanism of heart, kidney, and liver toxicity.

## 10 Synthesis: comparative conclusion

Owing to the inherent pathway heterogeneity of ferroptosis regulation, the multi-target, multi-pathway nature is a core characteristic of matrine as a natural product. Based on the dual complexity of ferroptosis and matrine, matrine regulates ferroptosis in different disease models by intervening in distinct upstream signals or effector molecules, making it difficult to explain all its effects through a single unified pathway. In septic myocardial injury, matrine inhibits ferroptosis via the GPX4/ACSL4 axis. In atherosclerosis, the key mechanism involves REG1A-mediated activation of the PI3K/AKT/mTOR pathway to suppress ferroptosis. In EAE, the suppression of ferroptosis involves the SLC7A11/GPX4 axis. In cervical cancer, matrine induces ferroptosis through the Piezo1 pathway.

As shown in Table 3, conflicting findings regarding the biological activities of matrine primarily involve two domains: Nrf2 pathway regulation and dose-dependent biphasic responses. Regarding Nrf2 modulation, matrine exhibits marked context-dependent effects: it enhances Nrf2 nuclear translocation and upregulates downstream antioxidant and anti-ferroptotic effectors in inflammatory cardiovascular disease models, whereas in toxicity models using healthy organs, it suppresses Nrf2 nuclear translocation and transcriptional activity, thereby promoting ferroptosis. These divergent effects may stem from cell-type specificity, biphasic dose–response properties, or distinct disease microenvironments. This biphasic property necessitates precise dose adjustment. Organ-specific susceptibility further complicates risk assessment: the kidneys appear to be more vulnerable to matrine-induced ferroptosis, with marked tubular injury occurring at doses that only cause mild hepatic steatosis. However, direct comparative investigations under standardized experimental conditions remain scarce. From a translational perspective, these inconsistencies pose significant challenges for the clinical development of matrine and highlight critical gaps in the current preclinical evidence base.

**TABLE 3:** Matrine: a critical analysis of conflicting evidence.

Perspective		Evidence level	References	Research limitations
(1) Modulating the Nrf2 pathway				
Perspective A	Activating Nrf2 nuclear translocation and upregulating HO-1, NQO1, GPX4 and reducing oxidative stress and ferroptosis	A (well-controlled animal and cell studies)	Xiao et al. (2024); Zhao et al. (2025)	All data deriving from rodent models and cell lines; lacking human data; no long-term human efficacy studies
Perspective B	Suppressing Nrf2 nuclear translocation and transcriptional activity, downregulating xCT, GPX4, FSP1, promoting ferroptosis and causing toxicity	A (well-controlled animal and cell studies)	Wang et al. (2024); Xi Wang et al. (2023); Wang et al. (2024)	
(2) Dose-dependent biphasic effects				
Perspective A	Using low and moderate doses to provide therapeutic effects, with a meta-analysis of 24 rodent studies defining the optimal liver-protective range: 20–30 mg/kg/d for short duration (0.02–0.86 weeks)	A (systematic review and meta-analys)	Xiao et al. (2024); Feng et al. (2024)	The meta-analysis being limited to liver outcomes; routes and durations hindering clinical extrapolation; long-term safety studies being sparse
Perspective B	Administering high doses (≥75 mg/kg) to induce ferroptosis-mediated toxicity	A (animal models)	Xi Wang et al. (2023); Wang et al. (2024); Wang et al. (2024)	

Evidence level (adapted from CAMARADES/GRADE, for preclinical research): A = well-controlled animal studies, systematic reviews/meta-analyses; B = limited animal data or expert opinion.

## 11 Critical analysis

Current evidence indicates that matrine possesses the capacity to regulate ferroptosis. However, this conclusion is primarily based on preclinical studies (animal models, *in vitro* validation, without clinical ferroptosis endpoints). The data clearly demonstrate the phenotypic plasticity of matrine in inhibiting ferroptosis in oxidative stress tissues and inducing ferroptosis in tumor cells, but they do not demonstrate its target occupancy specificity, cross-species generalizability, or long-term safety. So, several limitations of this study should be acknowledged. Firstly, the vast majority of studies involve acute or short-term exposure (animal experiments typically employ continuous administration for 7–20 days), and there is a lack of safety data on chronic administration as well as unforeseen consequences that long-term ferroptosis regulation may bring (e.g., chronic Nrf2 inhibition leading to accumulation of oxidative damage, or long-term ferroptosis induction causing immunosuppression). Currently, no study has evaluated the safety and dynamic changes of ferroptosis after matrine administration for more than 3 months. Secondly, preclinical evidence mainly relies on cell models with limited representativeness (e.g., immortalized cell lines differ from primary cells or human tissues in metabolism and antioxidant capacity) and acute disease models in animals (which have pathological gaps compared with chronic, relapsing human diseases). Moreover, species differences in Nrf2 pathways, iron metabolism regulation, and disease pathophysiology limit translational validity. Thirdly, the regulation of ferroptosis pathways by matrine is mostly based on indirect evidence (primarily from inhibitor/agonist experiments), and there is a lack of direct target binding evidence identified through techniques such as drug affinity responsive target stability (DARTS), cellular thermal shift assay (CETSA), or surface plasmon resonance. Meanwhile, the crosstalk between ferroptosis and other forms of cell death remains to be clarified. Fourthly, clinical trial evidence is insufficient. Existing human studies are mostly phase I–II trials with small sample sizes, methodological inconsistencies, and short follow-up periods. Prospective, multicenter, randomized controlled trials are lacking to fully define the clinical feasibility and risk–benefit profile of matrine. Furthermore, the dose–response relationship has not been systematically studied, and there is a lack of pharmacokinetic modeling and dose optimization studies. Addressing the above limitations through longitudinal mechanistic studies, rigorous clinical validation, and organ-specific dose-ranging studies is essential for advancing matrine from the laboratory to clinical application. As shown in Table 4, we summarize the pharmacological evidence from the core studies on matrine-regulated ferroptosis included in this review. All primary studies included in this review used highly pure (≥95%) matrine monomers unless otherwise specified, rather than traditional botanical drugs extracts. This facilitates the elucidation of molecular mechanisms but fails to reflect the potential modulatory effects on ferroptosis resulting from multi-metabolite synergy or other metabolites present in crude extracts. At the same time, the lack of inter-batch quality control reduces the transparency of reproducibility.

**TABLE 4:** Summary of Pharmacological evidence for matrine-regulated ferroptosis.

Study	Disease model	Model type	Matrine dose range	Minimum effective concentration	Treatment	Positive control	Negative control	Matrine source
Zhao et al. (2025)	Atherosclerosis	*In vitro:* HUVECs (ox-LDL induced) *In vivo:* ApoE⁻/⁻ mice (high-fat diet)	*In vitro:* 0.5, 1, 2, 3, 4 mg/mL *In vivo:* 200 mg/kg/day	*In vitro:* 3 mg/mL *In vivo:* 200 mg/kg	*In vitro*: 24 h *In vivo*: 12 weeks (gavage every other day)	Erastin (10 μM), LY294002 (10 μM)	pcDNA3.1 empty vector	Meilun biotechnology
Xiao et al. (2023)	Sepsis-induced myocardial injury	*In vivo:* C57BL/6 mice (LPS induced)	25, 50 mg/kg	25 mg/kg	3 days pretreatment, then LPS injection; sacrificed 18 h post-injury	None	Saline	Not specified
Feng et al. (2024)	EAE	*In vivo:* C57BL/6 mice (MOG₃₅₋₅₅ immunization)	50 mg/kg/day	50 mg/kg/day	Daily i.p. from day 0–25 post-immunization	None	Saline	Shanghai yuanye bio-technology
Jin et al. (2023)	SAP-ALI	*In vitro:* BEAS-2B, MLE-12 cells (LPS induced) *In vivo:* WT and UCP2⁻/⁻ mice (caerulein + LPS)	*In vivo:* 200 mg/kg (single i.p. 1 h before modeling) *In vitro:* Not specified (LPS 1 μg/mL)	*In vivo:* 200 mg/kg	*In vivo:* sacrificed 24 h after modeling *In vitro:* LPS for 24 h	None	Saline (*in vivo*), NC siRNA (*in vitro*)	Sigma-Aldrich, M5319
Jin et al. (2024)	Cervical cancer	*In vitro:* SiHa cells *In vivo:* CB17 SCID mice (xenograft)	*In vitro:* 0.25, 0.5, 1.0, 2.0 mg/mL *In vivo:* 25, 50, 75 mg/kg (i.p. Every other day)	*In vitro:* 1.0 mg/mL (colony formation) *In vivo:* 75 mg/kg	*In vitro:* 24 h *In vivo:* 22 days	Cisplatin (2 mg/kg), Ferrostatin-1 (4 μM)	PBS	≥97% purity (Meilun biotechnology)
Wang et al. (2024) (cardiotoxicity)	Cardiotoxicity	*In vitro:* H9c2 rat cardiomyoblasts	1, 1.5, 2 mg/mL	1 mg/mL	24 h	Fer-1 (20 nM), RSL3 (2 μM), DMF (3 μM), SS (2 μM), CoQ10 (3 μM)	PBS, si-NC	Solarbio, IM0060
Wang et al. (2023)	Hepatotoxicity	*In vitro:* L02 cells *In vivo:* BALB/c mice	*In vitro:* 0.5, 1, 1.5 mg/mL *In vivo:* 75, 100 mg/kg	*In vitro:* 0.5 mg/mL *In vivo:* 75 mg/kg	*In vitro:* 24 h *In vivo:* 15 consecutive days	Fer-1 (1 μM *in vitro*, 5 mg/kg *in vivo*), t-BHQ (0.5 μM/10 mg/kg), SS (1 μM/0.1 mg/kg)	Saline/PBS	Aladdin, SM8130
Wang et al. (2024) (nephrotoxicity)	Nephrotoxicity	*In vitro:* NRK-52E cells *In vivo:* BALB/c mice	*In vitro:* 1 mg/mL *In vivo:* 100 mg/kg	*In vitro:* 1 mg/mL *In vivo:* 100 mg/kg	*In vitro:* 24 h *In vivo:* 20 consecutive days	Fer-1 (5 nM *in vitro*, 5 mg/kg *in vivo*), RSL3 (20 mM), SS (protective agent)	PBS, si-con	Solarbio, IM0060

## 12 Research gaps and technological innovations

The systematicity and integration of current research also require further enhancement. At present, traditional approaches in cell and molecular biology are predominantly adopted. The introduction of multi-omics technologies, such as metabolomics and proteomics, is expected to provide a more comprehensive view of biological variation, thereby facilitating a deeper understanding of matrine’s network of action. Individualized dosing strategies should be explored, with personalized doses developed based on patient genetic background (e.g., Nrf2 gene polymorphisms), metabolic characteristics (e.g., CYP450 activity), and disease stage. Nano-targeted delivery systems should be developed, as matrine has low oral bioavailability and a short half-life. In the future, organ-targeted nanoformulations (e.g., myocardial-targeted liposomes, lung-targeted polymeric micelles, tumor microenvironment-responsive nanoparticles) should be developed to increase the concentration at the lesion site and reduce normal tissue exposure. In parallel, the research perspective remains relatively confined within the domains of pharmacology and pathology. Actively incorporating novel concepts and tools from cross-disciplinary fields such as materials science and bioinformatics may yield unexpected insights to help overcome existing bottlenecks in matrine research. Future efforts should be deepened in the following areas: refining mechanistic analyses, optimizing clinical trial designs, developing individualized toxicity management strategies, and embracing interdisciplinary collaboration. Through such approaches, the translation of matrine from the laboratory to the bedside can be advanced more steadily. Prior to this, translational claims of matrine as a ferroptosis-targeting agent should be regarded as mechanism-driven scientific hypotheses rather than clinical-ready therapeutic strategies.

## 13 Discussion

### 13.1 Current patents and future directions

A total of 3,360 Chinese patents related to matrine have been retrieved from CNKI(www.cnki.net) to date, along with 73 patents from other countries, including 25 patents from the United States, 16 patents from South Korea, 13 patents from Europe, 10 patents from Japan, four patents from Canada, two from patents the United Kingdom, one patent from Germany, one patent from Russia, and one patent from Australia. Compared to Matrine itself, the number of patents related to its derivatives is significantly lower, with 168 Chinese patents and three patents from other countries, including two patents from Europe and one patent from the United States. This reflects the “source innovation” stage of in-depth chemical modification of matrine to optimize its pharmacological properties and patent protection, and there is still huge room for improvement in the overall research and development activity. Derivative patents are a key step in the generation of drug candidates and the protection of intellectual property rights. The relatively limited number of derivative patents suggests that the transformation efficiency from “natural molecules” to “optimized drug candidates” needs to be strengthened. At present, this field is in a critical stage of transition from “extensive application exploration” to “precision drug development”. We encourage interdisciplinary collaboration (e.g., synthetic chemistry, structural biology, computational pharmacy, and clinical medicine) to pool resources for rational design and efficient screening of derivatization, which is the key to enhance their global competitiveness.

Future research needs to focus on screening the candidate molecules or protocols with the most promising translation prospects from these patents, and carry out rigorous preclinical validation and high-quality randomized controlled clinical trials in accordance with international standards to generate solid evidence of efficacy and safety. Thus, bridging the gap between extensive patent filings and globally recognized therapeutic breakthroughs. This is essential for translating traditional medical wisdom into innovative therapies that meet modern medical standards.

### 13.2 Traditional Chinese medicine: integration with modern therapeutic paradigms

As an integrated medical system with multiple factors to regulate multiple targets, traditional Chinese medicine is also increasingly demonstrating its special significance in today’s chronic disease control practice. The core concept, the “holistic view,” and the principle of “syndrome differentiation and treatment”, provide new strategies for intervention and new perspectives in the diagnosis, treatment, and prevention of modern diseases, which could potentially solve some problems faced by modern medicine. Natural alkaloids, represented by matrine, are typical examples of modern traditional Chinese medicine research, and their research proves the feasibility and effectiveness of the research approach of “active metabolites-molecular targets” in traditional Chinese medicine. This paper systematically explains how matrine regulates the key pathway of ferroptosis and exerts therapeutic potential across a variety of diseases, providing a new perspective for understanding its broad-spectrum efficacy.

### 13.3 Navigating toxicity: challenges and mitigation strategies

The therapeutic potential of matrine must be balanced against documented safety concerns, including dose-dependent cardiotoxicity, hepatotoxicity, and nephrotoxicity. These adverse effects appear mechanistically linked to antioxidant system inhibition and ferroptosis induction in healthy tissues, necessitating meticulous attention to dosing protocols and treatment duration in clinical applications. At the same time, potential drug interactions warrant consideration. Matrine is primarily metabolized by hepatic cytochrome P450 enzymes, particularly CYP3A4 and CYP2D6. Co-administration with CYP3A4 inhibitors (e.g., azole antifungals, macrolide antibiotics, grapefruit juice) could elevate matrine exposure, potentially precipitating toxicity. Conversely, CYP3A4 inducers (e.g., rifampicin, phenytoin) may reduce matrine levels and lead to therapeutic failure. Pharmacodynamically, combining matrine with other Nrf2 inhibitors (e.g., brusatol) could worsen its organ toxicity, while co-administration with Nrf2 activators (e.g., dimethyl fumarate, sulforaphane) might reduce toxicity but could also antagonize matrine’s pro-ferroptotic anticancer effect. None of these potential Drug–drug interactions (DDIs) have been systematically studied *in vivo*. We recommend DDI screening using human liver microsomes, hepatocytes, and confirmatory animal models before clinical combination trial is initiated.

Recent preclinical investigations indicate that co-administration with Nrf2 activators or selenium supplementation may attenuate matrine-induced toxicity, offering promising pharmacological strategies to enhance therapeutic indices. However, current evidence derives primarily from *in vitro* and animal studies; clinical efficacy and long-term safety profiles of these protective interventions remain undefined. Furthermore, existing toxicity mitigation approaches use standardized protocols that inadequately account for inter-individual variability in drug metabolism and pharmacodynamics. The development of personalized toxicity management strategies—incorporating pharmacogenomic profiling and organ-specific biomarker monitoring—represents a critical priority for future investigations.

## 14 Conclusion

This review explores the regulation of ferroptosis by matrine, through which matrine achieves organ protection or therapeutic effects in various diseases. Current treatments for many diseases face the following common challenges: single drug targets that fail to block multiple pathological pathways, drug resistance and toxicity, and a lack of effective strategies for organ protection. The unique value of matrine lies in the fact that it is not a single-target inhibitor, but rather modulates ferroptosis-related pathways to inhibit lipid peroxidation, regulate iron metabolism, and restore the antioxidant system in multiple disease models.

As a potential drug candidate, the most realistic path for matrine is to serve as a combination therapy or an organ-protective agent for precise application in high-risk populations. We recommend prioritizing indications with clear mechanisms and urgent clinical needs for breakthrough progress. Septic myocardial injury and severe acute pancreatitis-associated acute lung injury currently lack specific therapeutic agents. Matrine has shown cardiopulmonary protective effects in animal models by inhibiting ferroptosis, and the involved pathways (PI3K/AKT, UCP2/SIRT3) are relatively well-defined. Small-scale exploratory clinical trials are recommended, with cardiac biomarkers and lung injury scores as primary endpoints. The mechanism by which matrine induces ferroptosis via Piezo1 differs from conventional chemotherapy, and its combination with cisplatin synergistically enhances the tumor inhibition rate. A phase I/II trial is recommended to enroll patients with platinum-resistant or relapsed disease to evaluate the safety and objective response rate of matrine combined with cisplatin. Furthermore, tumor microenvironment-responsive prodrugs should be developed in parallel, with nanoparticles designed to release active matrine only at the tumor site, thereby achieving separation of efficacy and toxicity.

Natural metabolites have potential relevance in chronic disease management and precision medicine. Future research should prioritize mechanistic elucidation, rigorous toxicity profiling, and methodologically robust clinical evaluation. Through integration of advanced technological platforms and interdisciplinary collaboration, matrine may be positioned within evidence-based therapeutic frameworks, maximizing clinical utility while reflecting its traditional medicinal context.

## Glossary

4-HNE: 4-Hydroxynonenal
**ACSL4**: Acyl-CoA Synthetase Long Chain Family Member 4
**ALI**: Acute Lung Injury
**ALT/AST**: Alanine Aminotransferase/Aspartate Aminotransferase
**ARDS**: Acute Respiratory Distress Syndrome
**AS**: Atherosclerosis
**AUC**: Area Under the Curve
**Bax/Bcl2**: BCL2-Associated X Protein/B-cell Lymphoma 2
**Bcl-xL**: B-cell lymphoma-extra large
**BEAS-2B**: A human normal bronchial epithelial cell line
**BUN**: Blood Urea Nitrogen
**ChiCTR**: Chinese Clinical Trial Registry
**CNS**: Central Nervous System
**CPT1A**: Carnitine Palmitoyltransferase 1A
**CRE**: cAMP Response Element
**Cr**: Creatinine
**CTH**: Cystathionine Gamma-Lyase
**CTX**: Chrysotoxine
**CYP450**: Cytochrome P450
**CVD**: Cardiovascular Diseases
**DEG**: Differentially Expressed Gene
**DMF**: Dimethyl Fumarate
**DMT1**: Divalent Metal Transporter 1
**DNA**: Deoxyribonucleic Acid
**DOX**: Doxorubicin
**EAE**: Experimental Autoimmune Encephalomyelitis
**EGFR**: Epidermal Growth Factor Receptor
**EMT**: Epithelial-Mesenchymal Transition
**ERK1/2**: Extracellular Signal-Regulated Kinase 1/2
**ERS**: Endoplasmic Reticulum Stress
**ESR**: Erythrocyte Sedimentation Rate
**Fer-1**: Ferrostatin-1
**FLS**: Fibroblast-like Synoviocytes
**FSP1**: Ferroptosis Suppressor Protein 1
**FTH**: Ferritin Heavy Chain
**FTH1**: Ferritin Heavy Chain 1
**GO**: Gene Ontology
**GPX4**: Glutathione Peroxidase 4
**GSEA**: Gene Set Enrichment Analysis
**GSH**: Glutathione
**GSVA**: Gene Set Variation Analysis
**H9c2**: A rat cardiomyoblast cell line
**HBV**: Hepatitis B Virus
**HMGB1**: High Mobility Group Box 1
**HO-1**: Heme Oxygenase-1
**HUVEC**: Human Umbilical Vein Endothelial Cells
**ICB**: Immune Checkpoint Blockade
**IFN-β**: Interferon-beta
**IL**: Interleukin
**JAK/STAT**: Janus Kinase/Signal Transducer and Activator of Transcription
**K562**: A human chronic myeloid leukemia cell line
**KEGG**: Kyoto Encyclopedia of Genes and Genomes
**LCSCs**: Liver Cancer Stem Cells
**LDH**: Lactate Dehydrogenase
**LOX**: Lipoxygenase
**LPCAT3**: Lysophosphatidylcholine Acyltransferase 3
**LPO**: Lipid Peroxidation products
**LPS**: Lipopolysaccharide
**LVEF**: Left Ventricular Ejection Fraction
**LVFS**: Left Ventricular Fractional Shortening
**m6A**: N6-methyladenosine
**MASM**: A Matrine Derivative
**MDA**: Malondialdehyde
**METTL3**: Methyltransferase-like 3
**MIRI**: Myocardial Ischemia-Reperfusion Injury
**MLE-12**: A mouse lung epithelial cell line
**MLKL**: Mixed Lineage Kinase Domain-Like Pseudokinase
**MODS**: Multiple Organ Dysfunction Syndrome
**MPO**: Myeloperoxidase
**MSCs**: Mesenchymal Stem/Stromal Cells
**mTOR**: Mechanistic Target of Rapamycin
**MT**: Matrine
**NAC**: N-Acetylcysteine
**NETs**: Neutrophil Extracellular Traps
**NF-κB**: Nuclear Factor Kappa-Light-Chain-Enhancer of Activated B Cells
**NGF**: Nerve Growth Factor
**NLRP3**: NLR Family Pyrin Domain Containing 3
**NQO1**: NAD(P)H Quinone Dehydrogenase 1
**Nrf2**: Nuclear Factor Erythroid 2-Related Factor 2
**NSCLC**: Non-Small Cell Lung Cancer
**OECD**: Organisation for Economic Co-operation and Development
**OLG**: Oligodendrocyte
**OMT**: Oxymatrine
**ox-LDL**: Oxidized Low-Density Lipoprotein
**p38 MAPK**: p38 Mitogen-Activated Protein Kinase
**PGC1α**: Peroxisome Proliferator-Activated Receptor Gamma Coactivator 1-alpha
**PGE2**: Prostaglandin E2
**PI3K/AKT/mTOR**: Phosphoinositide 3-Kinase/Protein Kinase B/Mechanistic Target of Rapamycin
**Piezo1**: A mechanosensitive ion channel protein
**PLOOHs**: Phospholipid Hydroperoxides
**PTENP1/MIR-106B-5P**: PTENP1 Pseudogene/MicroRNA-106B-5P
**PTGS2**: Prostaglandin-Endoperoxide Synthase 2
**PTPN2/JNK/SREBP2**: Protein Tyrosine Phosphatase Non-Receptor Type 2/c-Jun N-terminal Kinase/Sterol Regulatory Element-Binding Protein 2
**PUFAs**: Polyunsaturated Fatty Acids
**RAGE**: Receptor for Advanced Glycation End Products
**rDNA**: Ribosomal DNA
**REG1A**: Regenerating Family Member 1 Alpha
**RLE-6TN**: A rat lung epithelial cell line
**ROS**: Reactive Oxygen Species
**RSL3**: A GPX4 inhibitor and ferroptosis inducer
**SAP**: Severe Acute Pancreatitis
**SAP-ALI**: Severe Acute Pancreatitis-Associated Acute Lung Injury
**Scr**: Serum Creatinine
**SIRT3**: Sirtuin 3
**SI-ALI**: Sepsis-Induced Acute Lung Injury
**SIRS**: Systemic Inflammatory Response Syndrome
**SLC7A11**: Solute Carrier Family 7 Member 11
**SPH/SPHK/S1P1**: Sphingosine/Sphingosine Kinase/Sphingosine-1-Phosphate Receptor 1
**SS**: Sodium Selenite
**TAMs**: Tumor-Associated Macrophages
**Tfr**: Transferrin Receptor
**TFR1**: Transferrin Receptor 1
**TNF-α**: Tumor Necrosis Factor-alpha
**TRIM3**: Tripartite Motif Containing 3
**UCP2**: Uncoupling Protein 2
**WM130**: A matrine derivative
**xCT**: Cystine/Glutamate Antiporter
**XIAP**: X-Linked Inhibitor of Apoptosis Protein
**ZEB1**: Zinc Finger E-Box Binding Homeobox 1
